# Mitochondrial unfolded protein response transcription factor ATFS-1 promotes longevity in a long-lived mitochondrial mutant through activation of stress response pathways

**DOI:** 10.1186/s12915-018-0615-3

**Published:** 2018-12-18

**Authors:** Ziyun Wu, Megan M. Senchuk, Dylan J. Dues, Benjamin K. Johnson, Jason F. Cooper, Leira Lew, Emily Machiela, Claire E. Schaar, Heather DeJonge, T. Keith Blackwell, Jeremy M. Van Raamsdonk

**Affiliations:** 1 Research Division, Joslin Diabetes Center, Boston, MA USA; 2 Department of Genetics, Harvard Medical School, Boston, MA USA; 3 Harvard Stem Cell Institute, Cambridge, MA USA; 4 Laboratory of Aging and Neurodegenerative Disease, Center for Neurodegenerative Science, Van Andel Research Institute, Grand Rapids, MI USA; 5 Bioinformatics and Biostatistics Core, Van Andel Research Institute, Grand Rapids, MI USA; 6 Department of Neurology and Neurosurgery, McGill University, Montreal, Quebec Canada; 7 Metabolic Disorders and Complications Program, and Brain Repair and Integrative Neuroscience Program, Research Institute of the McGill University Health Centre, Montreal, Quebec Canada

**Keywords:** Aging, Lifespan, Mitochondria, *C. elegans*, Mitochondrial unfolded protein response, ATFS-1, *clk-1*, *isp-1*, *nuo-6*, Genetics

## Abstract

**Background:**

The mitochondrial unfolded protein response (mitoUPR) is a stress response pathway activated by disruption of proteostasis in the mitochondria. This pathway has been proposed to influence lifespan, with studies suggesting that mitoUPR activation has complex effects on longevity.

**Results:**

Here, we examined the contribution of the mitoUPR to the survival and lifespan of three long-lived mitochondrial mutants in *Caenorhabditis elegans* by modulating the levels of ATFS-1, the central transcription factor that mediates the mitoUPR. We found that *clk-1*, *isp-1*, and *nuo-6* worms all exhibit an ATFS-1-dependent activation of the mitoUPR. While loss of *atfs-1* during adulthood does not affect lifespan in any of these strains, absence of *atfs-1* during development prevents *clk-1* and *isp-1* worms from reaching adulthood and reduces the lifespan of *nuo-6* mutants. Examining the mechanism by which deletion of *atfs-1* reverts *nuo-6* lifespan to wild-type, we find that many of the transcriptional changes present in *nuo-6* worms are mediated by ATFS-1. Genes exhibiting an ATFS-1-dependent upregulation in *nuo-6* worms are enriched for transcripts that function in stress response and metabolism. Consistent, with this finding, loss of *atfs-1* abolishes the enhanced stress resistance observed in *nuo-6* mutants and prevents upregulation of multiple stress response pathways including the HIF-1-mediated hypoxia response, SKN-1-mediated oxidative stress response and DAF-16-mediated stress response.

**Conclusions:**

Our results suggest that in the long-lived mitochondrial mutant *nuo-6* activation of the mitoUPR causes *atfs-1-*dependent changes in the expression of genes involved in stress response and metabolism, which contributes to the extended longevity observed in this mutant. This work demonstrates that the mitoUPR can modulate multiple stress response pathways and suggests that it is crucial for the development and lifespan of long-lived mitochondrial mutants.

**Electronic supplementary material:**

The online version of this article (10.1186/s12915-018-0615-3) contains supplementary material, which is available to authorized users.

## Background

The mitochondrial unfolded protein response (mitoUPR) is a stress response pathway that enables the mitochondria to communicate with the nucleus to upregulate mitochondrial chaperones and alter metabolism in response to mitochondrial stresses [1, 2]. This response is controlled by ATFS-1 (activating transcription factor associated with stress-1) in *Caenorhabditis elegans* [3] and ATF5 in mammals [4]. The ATFS-1 protein has both a mitochondria targeting sequence and a nuclear localization signal. Under normal conditions, ATFS-1 is imported into the mitochondria and degraded by the Lon protease [3]. When the mitochondria is stressed (e.g., by exposure to ROS [5], disrupted stoichiometry of the subunits of the electron transport chain [2], or mutations affecting mitochondria function [6]), the import of ATFS-1 into the mitochondria is prevented, allowing ATFS-1 to travel to the nucleus where it upregulates the expression of mitochondrial chaperones, various detoxification enzymes and metabolic enzymes [7].

In addition to ATFS-1, there are multiple other proteins involved in the mitoUPR including the mitochondrial matrix protease ClpP, the mitochondrial matrix peptide exporter HAF-1, the small ubiquitin-like protein, UBL-5, and the transcription factor DVE-1 [8–10]. Under conditions of mitochondrial stress, ClpP cleaves mitochondrial proteins, which are exported by HAF-1. These peptides have been proposed to inhibit the mitochondrial import of ATFS-1 [7], which accumulates in the cytoplasm and then travels to the nucleus where it acts in a complex with UBL-5 and DVE-1 to mediate the transcriptional changes associated with the mitoUPR. Among other changes, this includes increased expression of mitochondrial chaperone proteins, such as HSP-6, that help to re-establish proteostasis in the mitochondria.

Based on the observation that the mitoUPR is activated by knockdown of cytochrome c oxidase-1 (CCO-1) [2] and the fact that RNAi against *cco-1* increases lifespan [11], a role for the mitoUPR in longevity was explored by knocking down the expression of required components of the mitoUPR in long-lived worms with mildly impaired mitochondrial function. It was found that knocking down the expression of *ubl-5* reduces the lifespan of two long-lived mitochondrial mutants called *clk-1* and *isp-1*, but does not affect the lifespan of wild-type worms [12]. Similarly, decreasing the expression of *dve-1* reduced *isp-1* lifespan, but also markedly decreased wild-type lifespan making this observation more difficult to interpret [12]. It was subsequently shown that knocking down the expression of the mitochondrial ribosomal protein S5 (*mrps-5*), which causes an imbalance in between nuclear- and mitochondrially encoded components of the electron transport chain, causes both the activation of the mitoUPR and increased lifespan [13]. Importantly, decreasing *ubl-5* levels with RNAi reduced the increase in lifespan caused by *mrps-5* knockdown [13]. Activation of the mitoUPR has also been implicated in the lifespan extension caused by different types of bacteria [14] possibly through elevated reactive oxygen species [15]. Combined, these studies suggested that the mitoUPR plays a role in specific longevity pathways.

However, a subsequent study has suggested that the relationship between the mitoUPR and longevity is more complicated. After completing an RNAi screen for genes that activate the mitoUPR, it was shown that only some of these genes increase lifespan, while others decrease it [16]. This indicates that activation of the mitoUPR is not sufficient to increase lifespan. It was also shown that RNAi against *cco-1* can still increase the lifespan of an *atfs-1* deletion mutant (*tm4525*) even though the *atfs-1* mutation prevented the activation of the mitoUPR (as measured with an *hsp-6* reporter strain) [16]. Similarly, RNAi against *atfs-1* prevented the activation of the mitoUPR in *isp-1* mutants but did not impact longevity. Combined, these results suggest that activation of the mitoUPR is not required for longevity that is induced by *cco-1* RNAi or *isp-1* mutation. Similarly, it was shown that the increase in lifespan resulting from RNAi against a putative cytochrome c oxidase (F29C4.2) was not affected by loss of *atfs-1* [17]. Thus, at present, the role of the mitoUPR in determining longevity remains unclear [18].

In this work, we explore the role of the mitoUPR in the lifespan of three long-lived mitochondrial mutants: *clk-1*, *isp-1*, and *nuo-6* [19–22]. These genes encode proteins involved in the mitochondrial electron transport chain including a hydroxylase required for the synthesis of ubiquinone, a subunit of complex III called the Rieske iron sulfur protein and a subunit of complex I, respectively. In *clk-1* and *isp-1* mutants, we find that the mitoUPR is not required during adulthood for their long lifespans, but is required for these worms to develop to adulthood, making it impossible to assess its contributions to longevity conclusively. Importantly, *nuo-6* worms do not require the mitoUPR to develop to adulthood, thereby providing the opportunity to assess the role of the mitoUPR in longevity. We find that in *nuo-6* worms the activation of the mitoUPR causes the upregulation of stress response genes and metabolic enzymes and that this results in increased resistance to multiple stresses and long lifespan.

## Results

### Mitochondrial unfolded protein response is activated in long-lived mitochondrial mutants

**Fig. 1:**
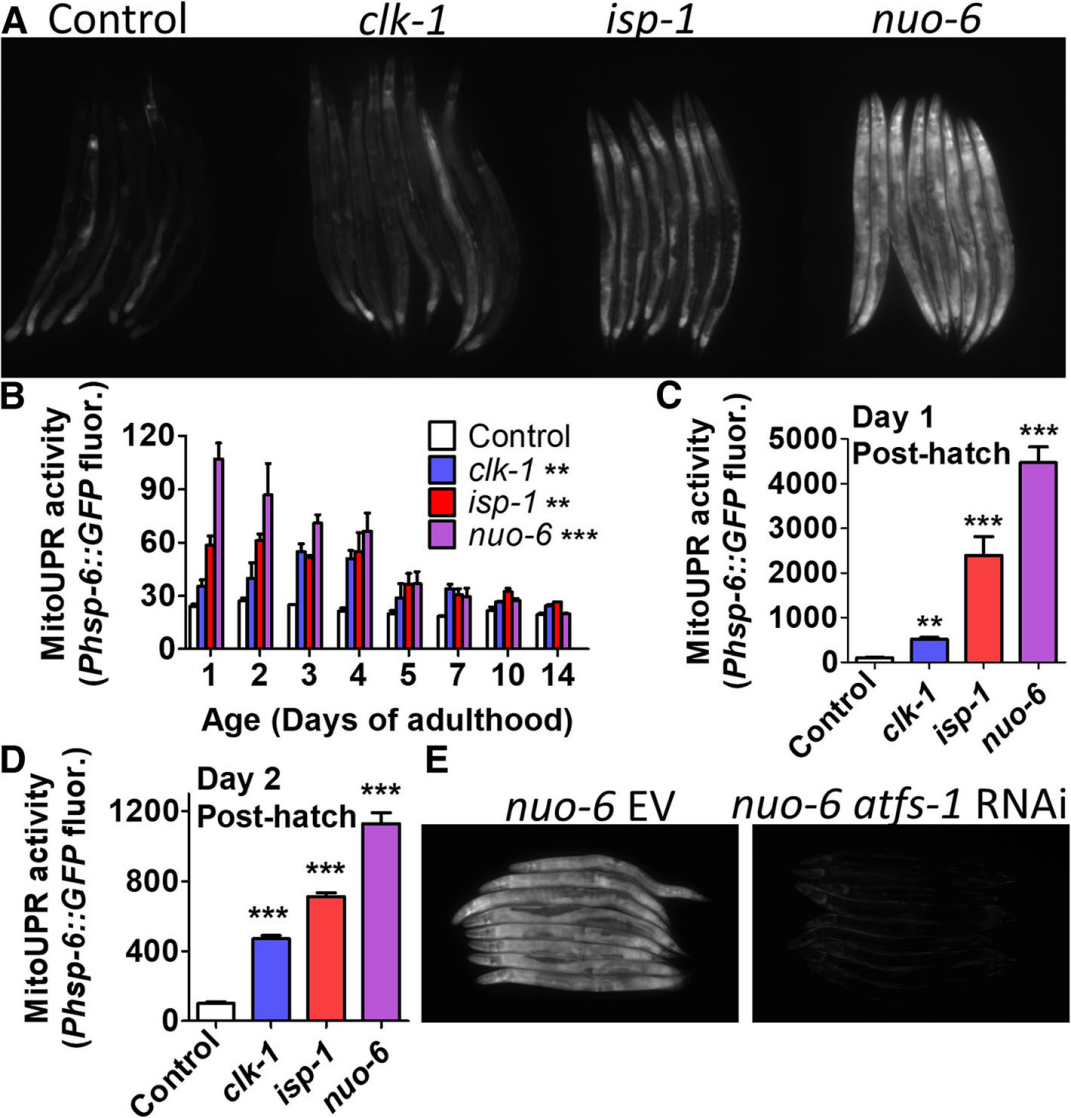
ATFS-1 dependent activation of the mitochondrial unfolded protein response (mitoUPR) in the long-lived mitochondrial mutants. **a** To monitor the activation of the mitoUPR, the mitochondrial mutants *clk-1*, *isp-1*, and *nuo-6* were crossed to the *Phsp-6::GFP* transcriptional reporter strain. All three strains shown increased GFP expression indicating activation of the mitoUPR. **b** A time course during adulthood reveals that the activation of the mitoUPR persists during early adulthood. **c**, **d** Similarly, all three mitochondrial mutants show activation of the mitoUPR as early as day 1 and day 2 after hatching. **e** The activation of the mitoUPR reporter is dependent on the ATFS-1 transcription factor, as *atfs-1* RNAi prevents GFP expression from *nuo-6;Phsp-6::GFP* worms*. atfs-1* knockdown was initiated at the parental L4 stage and fluorescence was examined in young adult progeny. Error bars indicate SEM. *p < 0.05, **p < 0.01, ***p < 0.001. See Additional file 5: Table S5 for details on N and replicates

In order to examine the role of the mitoUPR in the long lifespan of the mitochondrial mutants *clk-1*, *isp-1*, and *nuo-6*, we first sought to confirm that the mitoUPR is upregulated in these mutants [6, 12, 23]. To do this, we crossed the mitochondrial mutant strains to a reporter strain for the mitoUPR target gene *hsp-6* (*Phsp-6::GFP*) [2]*.* We found that *clk-1*, *isp-1*, and *nuo-6* worms all exhibit increased fluorescence compared to wild-type worms (Fig. 1a) indicating activation of the mitoUPR. We found that fluorescence from the mitoUPR reporter remained significantly increased until day 5 of adulthood (Fig. 1b). Since GFP-tagged proteins can have a half-life of multiple days, it is possible that fluorescence from the mitoUPR reporter strain during adulthood resulted from mitoUPR activity during development. To confirm that the mitoUPR remains activated in adulthood, we examined RNAseq data from *clk-1*, *isp-1*, and *nuo-6* mutants at the young adult stage [24]. We found that *hsp-6* mRNA is significantly increased in all three strains and is correlated with *atfs-1* mRNA levels (Additional file 1: Figure S1).

Next, we sought to determine whether the mitoUPR is also activated during larval development. We examined worms at 1 and 2 days after hatching and in both cases found that the mitoUPR is activated in *clk-1*, *isp-1*, and *nuo-6* worms (Fig. 1c, d). Finally, we wanted to determine whether the activation of the mitoUPR is dependent on ATFS-1. Accordingly, we treated *clk-1;Phsp-6::GFP*, *isp-1;Phsp-6::GFP*, and *nuo-6;Phsp-6::GFP* worms with RNAi targeting *atfs-1* and found that knocking down *atfs-1* prevented the induction of the mitoUPR (Fig. 1e, Additional file 1: Figure S2). Combined, these results demonstrate that the long-lived mitochondrial mutant strains exhibit an ATFS-1 dependent activation of the mitoUPR in development and early adulthood.

Since elevated ROS levels are sufficient to activate the mitoUPR [5, 25] and ROS levels have been shown to be increased in the long-lived mitochondrial mutants [26, 27], we next sought to determine the extent to which the activation of the mitoUPR is dependent on elevated ROS. To do this, we treated *nuo-6* worms with two different antioxidants, 10 mM vitamin C and 10 mM N-acetyl-cysteine, which have been previously shown to decrease the lifespan of long-lived mitochondrial mutants [26, 28]. Using the *Phsp-6::GFP* reporter fluorescence as a measurement of mitoUPR activity, we found that antioxidant treatment had no impact on mitoUPR activation (Additional file 1: Figure S3). This suggests that an elevation in ROS is not required for the *nuo-6* mutation to activate the mitoUPR. However, it is also possible that the antioxidant treatment did not reduce the levels of ROS sufficiently, or did not reduce the right population of ROS to observe an effect on mitoUPR activation.

### Mitochondrial unfolded protein response is required for survival and lifespan of long-lived mitochondrial mutants

To elucidate the role of the mitoUPR in the long-lifespan of *clk-1*, *isp-1*, and *nuo-6* worms, we examined how decreasing *atfs-1* expression affected their longevity. We used three different paradigms of increasing severity: *atfs-1* RNAi beginning in the experimental L4 generation, *atfs-1* RNAi beginning in the parental L4 generation and an *atfs-1(gk3094)* deletion mutation.

**Fig. 2:**
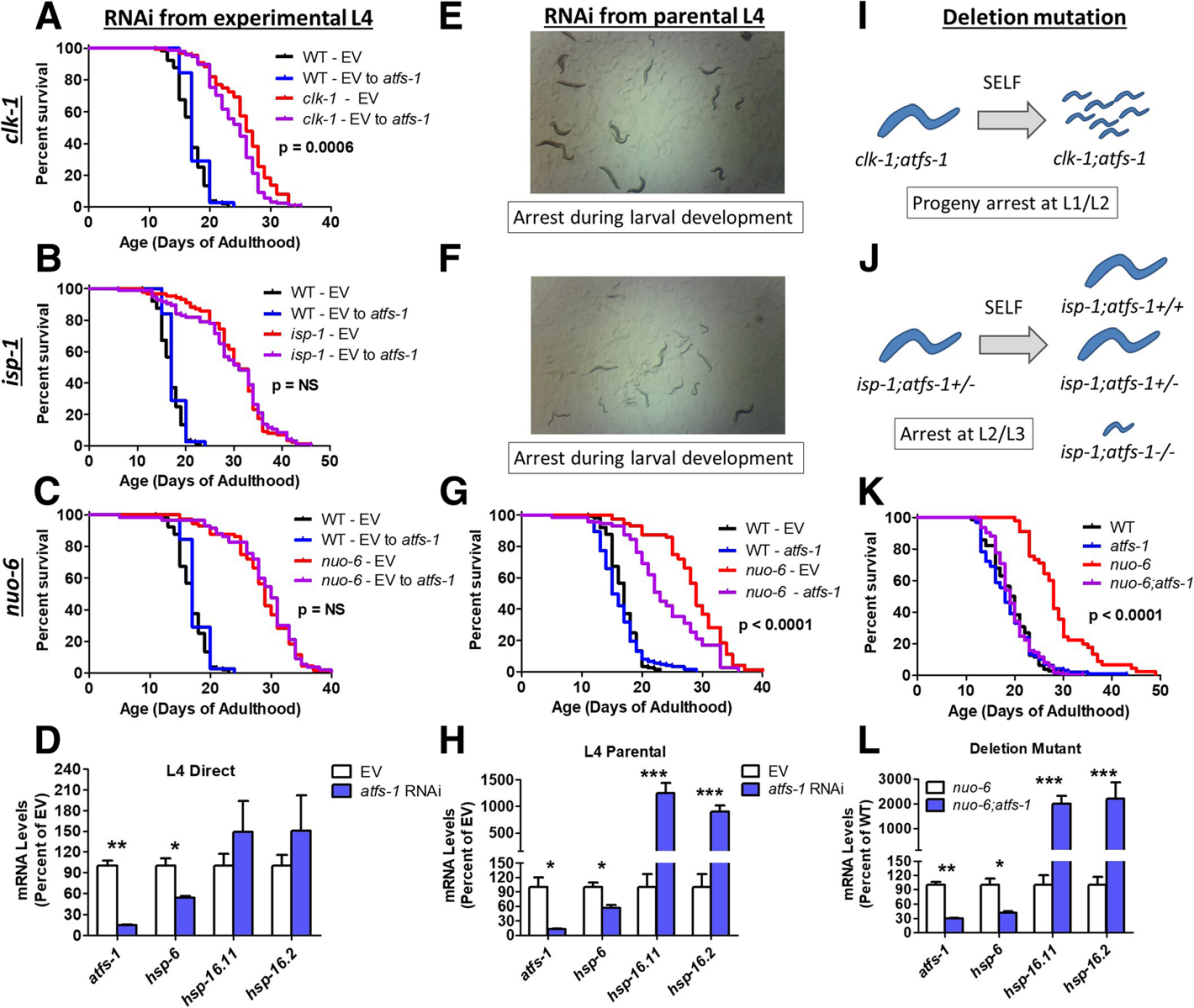
ATFS-1 is required for the survival and longevity of long-lived mitochondrial mutants. *atfs-1* levels were decreased using three paradigms of increasing severity: RNAi beginning from the experimental L4 generation (left column), RNAi beginning from the parental L4 generation (middle column) and deletion mutation (right column). Decreasing the expression of *atfs-1* beginning at the L4 stage of development had little or no effect on the lifespan of *clk-1* (**a**), *isp-1* (**b**), or *nuo-6* (**c**) worms, despite reducing the expression of *atfs-1* and the *atfs-1* target gene *hsp-6* (**d**). Decreasing the expression of *atfs-1* beginning in the parental generation resulted in developmental arrest in both *clk-1* (**e**) and *isp-1* (**f**) worms, while *nuo-6* worms grew to adulthood and showed a decreased lifespan (**g**). This treatment reduced *atfs-1* and *hsp-6* levels, and resulted in an upregulation of *hsp-16.11* and *hsp-16.2* (**h**). Deletion of *atfs-1* resulted in developmental arrest in *clk-1* (**i**) and *isp-1* (**j**) worms. In contrast, *nuo-6;atfs-1* worms are viable and the *atfs-1* deletion completely reverted *nuo-6* lifespan to wild-type (**k**). Deletion of *atfs-1* decreased *atfs-1* and *hsp-6* gene expression and increased the levels of *hsp-16.11* and *hsp-16.2* mRNA (**l**). Significance between red and purple lines is indicated. Error bars indicate SEM. *p < 0.05, **p < 0.01, ***p < 0.001. See Additional file 4: Table S4 for raw lifespan data. See Additional file 5: Table S5 for details on N and replicates

In the experimental L4 RNAi paradigm, worms are allowed to develop on NGM plates until the L4 stage of development, when they were transferred to *atfs-1* RNAi plates. Using this paradigm, we found that *atfs-1* RNAi caused a small decrease in *clk-1* lifespan (Fig. 2a), but did not affect the longevity of *isp-1* (Fig. 2b) or *nuo-6* worms (Fig. 2c). At day 2 of adulthood, this paradigm resulted in a significant decrease in *atfs-1* levels as well as the *atfs-1* target gene *hsp-6* (Fig. 2d), but did not affect the expression of cytosolic unfolded protein response targets *hsp-16.11* or *hsp-16.2* (which we found are robustly increased by *atfs-1* mutation—see Fig. 6).

In the parental L4 paradigm, worms are transferred to *atfs-1* RNAi plates at the L4 stage. When these worms become gravid adults, they are transferred to a second *atfs-1* RNAi plate and allowed to lay eggs for 24 h. The lifespan of the F1 progeny is then measured when the worms reach adulthood. Under this paradigm, we found that only wild-type and *nuo-6* worms are able to develop to adulthood, while *clk-1* and *isp-1* worms arrest during larval development (Fig. 2e, f; Additional file 1: Figures S4, S5), as has been observed previously [6]. We found that knocking down *atfs-1* beginning at the L4 stage in the parental generation significantly decreased *nuo-6* lifespan but did not affect wild-type longevity (Fig. 2g). This paradigm significantly reduced the expression levels of *atfs-1* and *hsp-6* to a similar extent as we observed in the experimental L4 paradigm. Unlike the experimental L4 paradigm, knocking down *atfs-1* beginning in the parental generation resulted in a significant increase in the expression of *hsp-16.11* and *hsp-16.2*, which are involved in the cytosolic unfolded protein response (also known as the heat shock response) (Fig. 2h). This suggests that there may be a greater degree of proteotoxic stress when *atfs-1* is knocked down in throughout life.

Finally, we examined the effect of an *atfs-1* deletion mutation on the lifespan of the mitochondrial mutants. We found that *clk-1;atfs-1* double mutants produce few progeny and that the progeny arrest at the L1 or L2 stage of development (Fig. 2i). Similarly, we found that *isp-1;atfs-1* double mutants arrest at the L2 or L3 stage (Fig. 2j). In contrast, *nuo-6;atfs-1* double mutants were found to be viable and fertile. Examining the lifespan of these double mutants revealed that the *atfs-1* deletion completely reverted *nuo-6* lifespan to wild-type but did not impact wild-type lifespan (Fig. 2k). The *atfs-1* deletion reduced expression of *atfs-1* and *hsp-6* and increased the expression of *hsp-16.11* and *hsp-16.2* (Fig. 2l). Combined, these results demonstrate that *atfs-1* is required for the development of *clk-1* and *isp-1* worms to adulthood and for the longevity of *nuo-6* worms. Loss of *atfs-1* does not decrease wild-type lifespan, and in the mitochondrial mutants, *atfs-1* is not needed during adulthood for normal lifespan.

Because we observed an effect of *atfs-1* RNAi on the lifespan of *nuo-6* mutants, we decided to examine other components of the mitoUPR including UBL-5, DVE-1 and HAF-1. We used the parental L4 paradigm in which we observed an effect of *atfs-1* RNAi in *nuo-6* worms. Under these conditions, we found that knocking down *dve-1* or *ubl-5* prevented *nuo-6* worms from developing to adulthood (Additional file 1: Figure S6).

Since *nuo-6* worms could develop to the L4 stage on *ubl-5* RNAi, we compared the survival of these worms to worms grown on empty vector RNAi. Consistent with our observations with *atfs-1* RNAi, we found that the L4 survival of *nuo-6* worms on *ubl-5* RNAi was markedly decreased compared to EV RNAi (Additional file 1: Figure S7A). On *haf-1* RNAi, *nuo-6* worms developed to adulthood and showed a similar lifespan to worms grown on EV RNAi (Additional file 1: Figure S7B). This suggests that the mechanism by which disruption of *nuo-6* activates the mitoUPR does not require HAF-1. As with the results of our lifespan studies, we found that *haf-1* RNAi had a different effect on activation of the cytosolic unfolded protein response compared to other components of the mitoUPR. While knockdown of *atfs-1*, *ubl-5*, or *dve-1* resulted in increased expression of the cytosolic unfolded protein response target genes, *hsp-16.11* and *hsp-16.12*, *haf-1* RNAi had no significant effect on the expression of either gene (Additional file 1: Figure S8). Again, this suggests that disruption of *haf-1* in the mitochondria affects the mitoUPR differently than disruption of the nuclear factors *atfs-1*, *dve-1*, and *ubl-5.*

Since deletion of the mitochondrial superoxide dismutase gene *sod-2* [29] and mild inhibition of mitochondrial function have both been proposed to increase lifespan through elevation of mitochondrial superoxide levels [26], we also examined the role of the mitoUPR in the longevity of *sod-2* worms. As in the long-lived mitochondrial mutants, we found that the mitoUPR is activated in *sod-2* mutants, as measured using the *Phsp-6::GFP* reporter strains (Additional file 1: Figure S9A). However, we found that knocking down the expression of *atfs-1* did not prevent *sod-2* worms from developing to adulthood, or impact their longevity (Additional file 1: Figure S9B, C). This indicates that the mechanisms underlying longevity in *sod-2* mutants are at least partially distinct from the long-lived mitochondrial mutants.

### Loss of ATFS-1 expression during development or adulthood alone does not shorten *nuo-6* lifespan

**Fig. 3:**
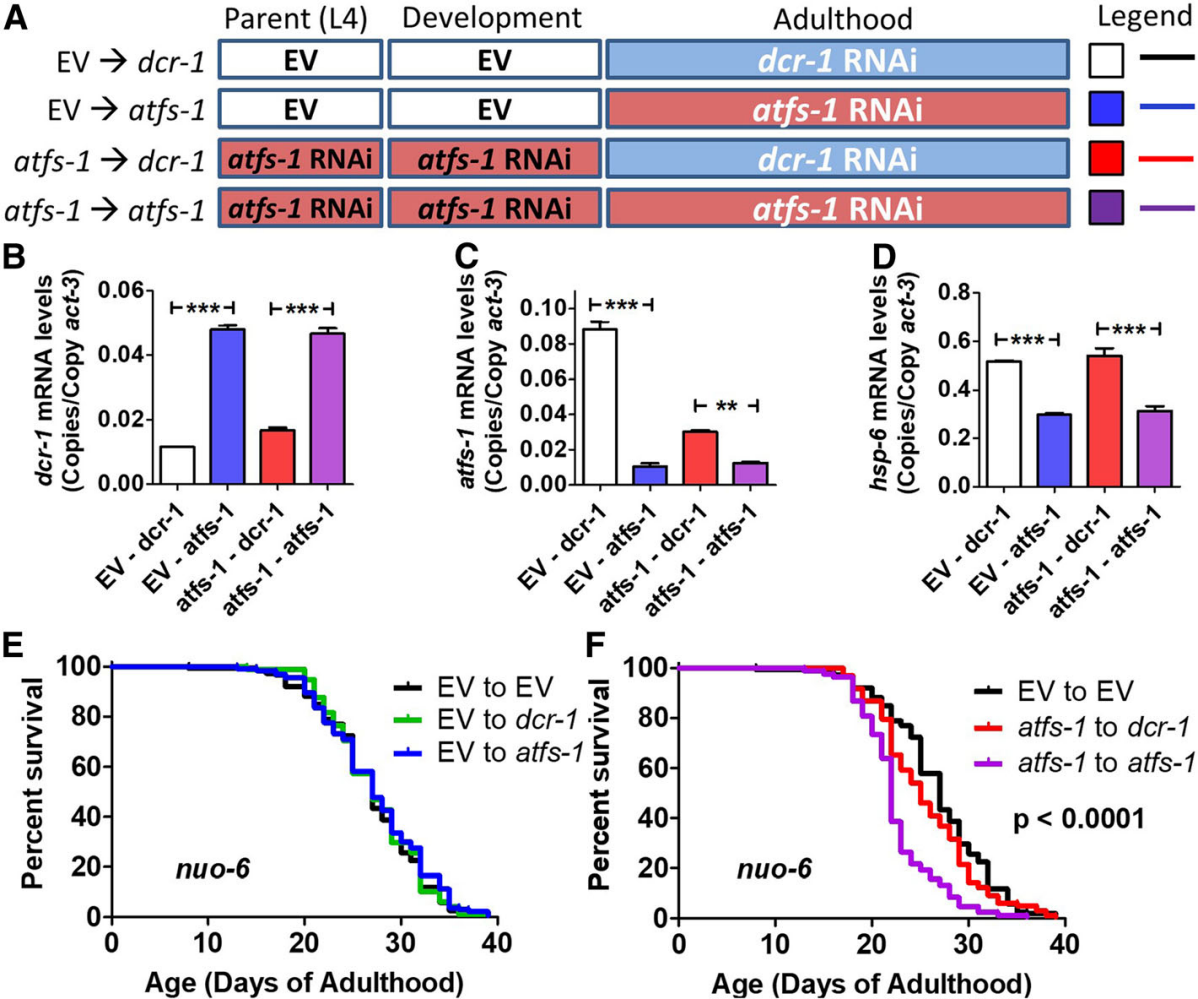
Loss of ATFS-1 during development or adulthood alone is not sufficient to decrease *nuo-6* longevity. **a** To explore the timing requirements for the role of ATFS-1 in *nuo-6* longevity and the activation of the mitoUPR, *nuo-6* worms were treated with RNAi against *atfs-1* for development only (*atfs-1 ➔ dcr-1*), adulthood only (EV ➔ *atfs-1*) or for both development and adulthood (*atfs-1* ➔ *atfs-1*). Quantitative real-time RT-PCR was used to confirm knockdown of *atfs-1*, *dcr-1* and the ATFS-1 target gene *hsp-6.* Treatment with *dcr-1* RNAi during adulthood effectively decreased the expression of *dcr-1* (**b**)*.* The levels of *atfs-1* mRNA were decreased by *atfs-1* RNAi during adulthood only or development and adulthood (**c**). Transfer from *atfs-1* RNAi to *dcr-1* RNAi resulted in a restoration of *atfs-1* mRNA levels towards EV control. Similarly, the levels of the *atfs-1* target gene *hsp-6* were decreased by *atfs-1* RNAi during adulthood only or development and adulthood (**d**). Transfer from *atfs-1* RNAi to *dcr-1* RNAi resulted in a complete restoration of *hsp-6* expression. **e** Knocking down *atfs-1* levels only during adulthood does not affect *nuo-6* lifespan. **f** Knocking down *atfs-1* only during development does not significantly reduce *nuo-6* lifespan. In contrast, *atfs-1* RNAi applied during both development and adulthood decreased *nuo-6* longevity. Error bars indicate SEM. **p < 0.01, ***p < 0.001. See Additional file 4: Table S4 for raw lifespan data. See Additional file 5: Table S5 for details on N and replicates

The fact that knocking down *atfs-1* during adulthood had little or no impact on lifespan suggests the possibility that ATFS-1 affects lifespan during development. Accordingly, we sought to determine whether knocking down *atfs-1* levels only during development would be sufficient to decrease lifespan. To do this, we utilized an approach where worms are grown on *atfs-1* RNAi during development and then transferred to RNAi against dicer (*dcr-1*) at adulthood [11, 12, 30]. By knocking down dicer, which is an essential component of the RNAi machinery, inhibition of *atfs-1* expression should be prevented allowing *atfs-1* levels to be restored. Worms were grown on empty vector or *atfs-1* RNAi beginning at the L4 developmental stage of the parental generation and then transferred to either *dcr-1* RNAi plates (to restore *atfs-1* expression) or *atfs-1* RNAi plates (to continue *atfs-1* knockdown) at adulthood (Fig. 3a).

Because *atfs-1* is absolutely required in *clk-1* and *isp-1* worms for development to adulthood, we completed these experiments, and all of the remaining experiments in *nuo-6* worms. We used quantitative real-time RT-PCR at day 2 of adulthood to examine the levels of *dcr-1*, *atfs-1*, and *hsp-6.* We found that transfer to *dcr-1* RNAi plates at adulthood effectively decreased the expression of *dcr-1* (Fig. 3b). The levels of *atfs-1* were efficiently knocked down by *atfs-1* RNAi and were significantly increased when transferred to *dcr-1* RNAi (Fig. 3c). Importantly, treatment with *atfs-1* RNAi during adulthood significantly reduced *hsp-6* expression, while *hsp-6* expression in worms grown on *atfs-1* RNAi during development and transferred to *dcr-1* RNAi at adulthood (*atfs-1* ➔ *dcr-1* paradigm) was equivalent to worms that were not treated with *atfs-1* RNAi (EV ➔ *dcr-1* paradigm) (Fig. 3d).

Having shown that *atfs-1* levels were being knocked down specifically during development, we next examined the resulting effect on lifespan. We found that knocking down *atfs-1* expression during adulthood (EV ➔ *atfs-1* paradigm) did not decrease the lifespan of *nuo-6* worms (Fig. 3e). Similarly, decreasing *atfs-1* expression during development alone (*atfs-1* ➔ *dcr-1* paradigm) was not sufficient to decrease *nuo-6* lifespan (Fig. 3f). Note that the lifespan of *nuo-6* worms treated with the *atfs-1* ➔ *dcr-1* paradigm showed significantly increased lifespan compared to worms treated with either the *atfs-1* ➔ *atfs-1* paradigm or *atfs-1* ➔ EV paradigm (Additional file 1: Figure S10), providing further evidence that the transfer to *dcr-1* RNAi was effective at restoring *atfs-1* expression (continued knockdown of *atfs-1* in *atfs-1* ➔ EV paradigm leads to decrease in lifespan). Thus, in order for *atfs-1* knockdown to decrease *nuo-6* lifespan, *atfs-1* levels had to be decreased during both development and adulthood.

### Loss of ATFS-1 affects physiologic rates and mitochondrial function of *nuo-6* worms

**Fig. 4:**
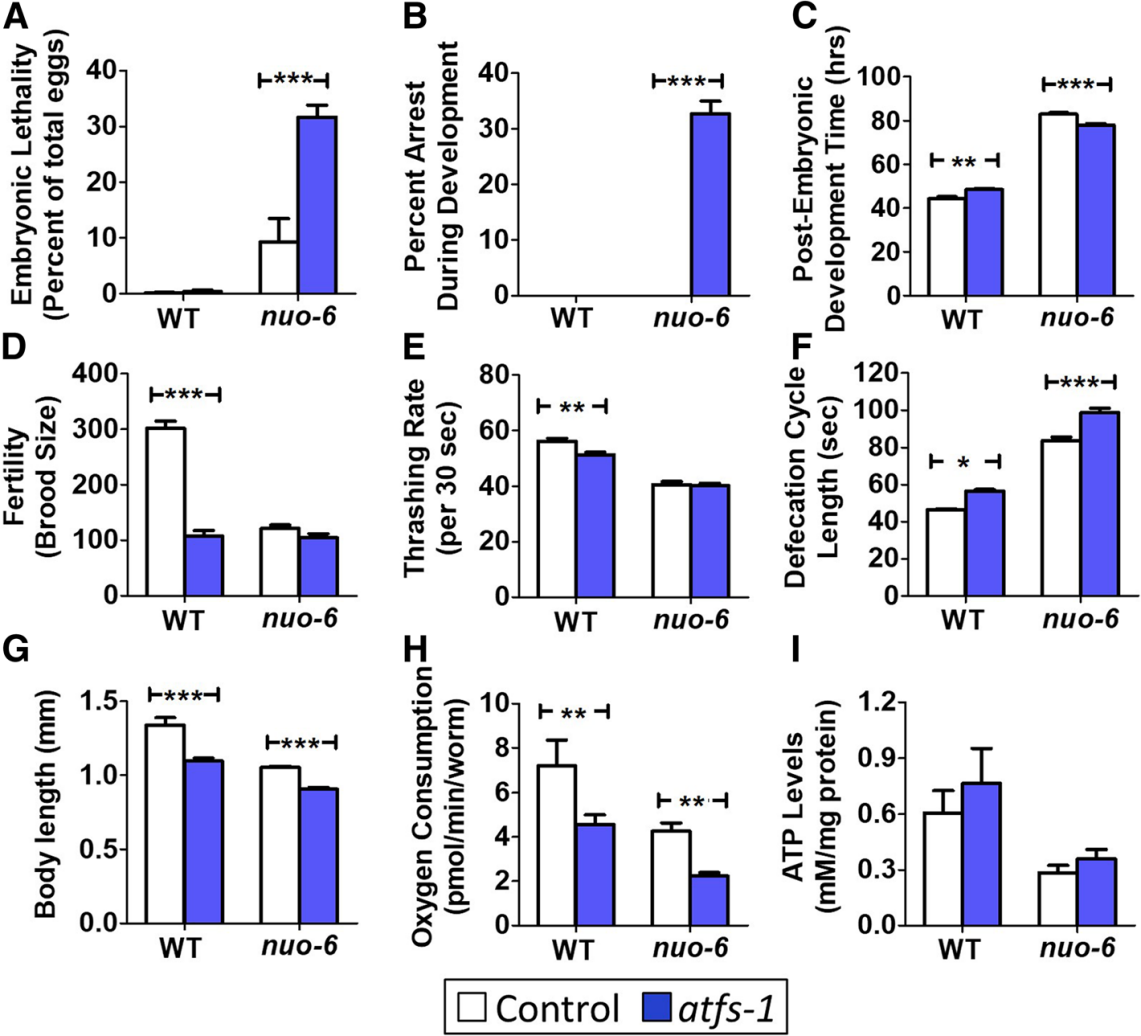
Loss of *atfs-1* alters physiologic rates in the *nuo-6* mitochondrial mutant. **a** *nuo-6* worms have increased embryonic lethality compared to wild-type worms, and lethality is further increased by deletion of *atfs-1*. **b** While all *nuo-6* worms develop to adulthood after hatching, a proportion of *nuo-6;atfs-1* worms arrest during larval development. **c** Deletion of *atfs-1* slows development in wild-type worms but accelerates development time in *nuo-6* worms. **d** Loss of *atfs-1* decreases brood size in wild-type worms but does not further decrease the low brood size in *nuo-6* worms. **e** The *atfs-1* deletion decreases the rate of movement in wild-type worms but does not exacerbate the slow movement of *nuo-6* worms. Loss of *atfs-1* causes a slow rate of defecation (**f**) decreased body length (**g**), and decreased oxygen consumption (**h**) in both wild-type and *nuo-6* worms, but does not significantly affect ATP levels (**i**). Error bars indicate SEM. *p < 0.05,**p < 0.01, ***p < 0.001. See Additional file 5: Table S5 for details on N and replicates

To begin to explore the mechanism by which loss of *atfs-1* reduces the lifespan of *nuo-6* worms, we compared physiologic rates and mitochondrial function in *nuo-6* and *nuo-6;atfs-1* worms. We found that the *atfs-1* mutation increased both embryonic lethality and developmental arrest specifically in *nuo-6* worms but not in wild-type worms (Fig. 4a, b). Of those worms that do develop to adulthood, *atfs-1* mutants develop more slowly than wild-type worms, while *nuo-6;atfs-1* worms reach adulthood in less time than *nuo-6* worms (Fig. 4c). Loss of *atfs-1* results in decreased brood size but does not further decrease the already low brood size of *nuo-6* worms (Fig. 4d). Similarly, *atfs-1* mutants have a slower thrashing rate than wild-type worms, but the mutation does not exacerbate the thrashing deficit present in *nuo-6* mutants (Fig. 4e). The loss of *atfs-1* results in a slower rate of defecation and decreased body length in both wild-type and *nuo-6* worms (Fig. 4f, g).

In examining mitochondrial function, we determined the rate of oxidative phosphorylation by measuring oxygen consumption per worm using an extracellular flux analyzer. We found that oxygen consumption per worm is decreased by the loss of *atfs-1* in both wild-type worms and *nuo-6* mutants (Fig. 4h). To determine if the rates of oxygen consumption resulted in altered levels of ATP, we used a lucigenin-based kit to measure ATP levels. We found that the *atfs-1* mutation did not decrease ATP levels in wild-type or *nuo-6* worms (Fig. 4i). To gain insight into the relationship between oxygen consumption and ATP levels, we examined the effect of the *atfs-1* mutation on ROS levels in wild-type and *nuo-6* worms. Consistent with the decreased levels of oxygen consumption, ROS levels as detected by dichlorofluorescein (DCF) fluorescence were significantly decreased by *atfs-1* deletion (Additional file 1: Figure S11). Combined, these results show that disruption of *atfs-1* negatively impacts worm physiologic rates and mitochondrial function, and this may at least partially explain the detrimental effect of the *atfs-1* mutation on *nuo-6* lifespan and the ability of *clk-1* and *isp-1* worms to develop to adulthood.

### Loss of ATFS-1 decreases resistance to multiple stresses

**Fig. 5:**
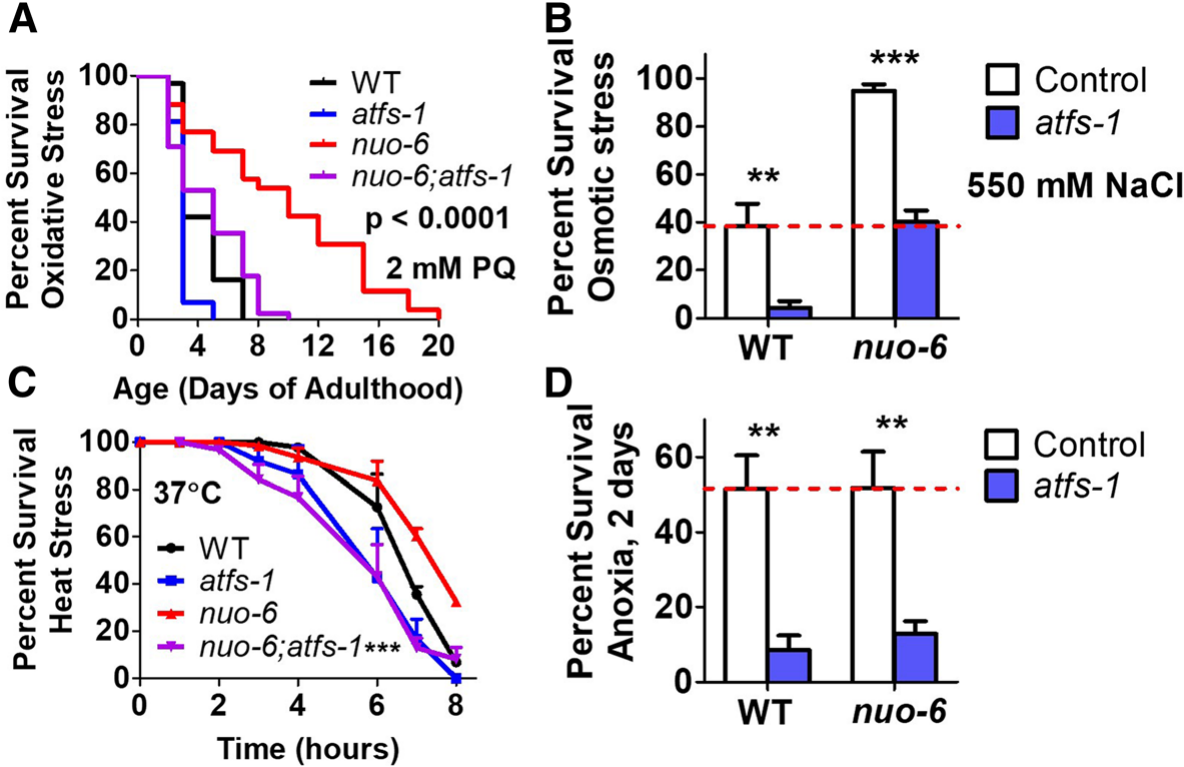
Loss of *atfs-1* reduces increased resistance to multiple stresses in long-lived *nuo-6* worms and decreases stress resistance in wild-type worms. **a** Sensitivity to oxidative stress was assessed by exposing worms to plates containing 2 mM paraquat (PQ). *nuo-6* worms showed increased resistance to oxidative stress, which was completely abolished by removal of *atfs-1.* **b** Sensitivity to osmotic stress was assessed by exposing worms to 550 mM NaCl and quantifying survival after 48 h. *nuo-6* worms exhibit increased survival compared to wild-type worms. Loss of *atfs-1* resulted in decreased survival in both wild-type and *nuo-6* worms. **c** To measure sensitivity to heat stress, worms were incubated at 37 °C and survival was monitored hourly. *nuo-6* worms exhibit increased survival compared to wild-type worms. In both cases, heat stress survival was decreased by loss of *atfs-1.* **d** Deletion of *atfs-1* causes increased sensitivity to anoxia in both wild-type and *nuo-6* worms. Error bars indicate SEM. In panels A and C, *p* value indicates significance between *nuo-6* and *nuo-6;atfs-1* worms. **p < 0.01, ***p < 0.001. See Additional file 5: Table S5 for details on N and replicates

Since the ability to resist multiple stresses has been associated with long life, we next examined the effect of the *atfs-1* mutation on stress resistance in *nuo-6* worms. Sensitivity to oxidative stress was assessed by exposing worms to 2 mM paraquat, a compound that acts to increase superoxide levels primarily in the mitochondria. We found that *nuo-6* worms have increased survival compared to wild-type worms and that the *atfs-1* mutation completely reverts their stress resistance to wild-type (Fig. 5a). *atfs-1* mutants also exhibited a small decrease in oxidative stress resistance compared to wild-type worms.

Sensitivity to osmotic stress was quantified by transferring worms to plates containing 550 mM NaCl (11X their normal salt concentration) and examining survival after 2 days. Again, we found that *nuo-6* worms have increased resistance to osmotic stress, which was decreased in the presence of the *atfs-1* mutation, and *atfs-1* mutants had decreased survival compared to wild-type worms (Fig. 5b).

In the heat stress assay, worms are exposed to 37 °C heat stress and survival is monitored hourly. *nuo-6* worms exhibited a mildly increased resistance to heat stress (Fig. 5c). Loss of *atfs-1* decreased the survival of both *nuo-6* and wild-type worms during heat stress (Fig. 5c).

Finally, we examined survival after 2 days of anoxia with a one-day recovery. We found that *nuo-6* worms had an equivalent survival to wild-type and that in both cases survival was decreased by the *atfs-1* mutation (Fig. 5d). Combined, these results indicate that ATFS-1 is important for survival against multiple forms of stress. The decrease in stress resistance in the *nuo-6;atfs-1* double mutant may at least partially explain the decrease in longevity compared to *nuo-6* mutants.

### *nuo-6* worms exhibit ATFS-1-dependent changes in expression of genes involved in stress response and metabolism

**Fig. 6:**
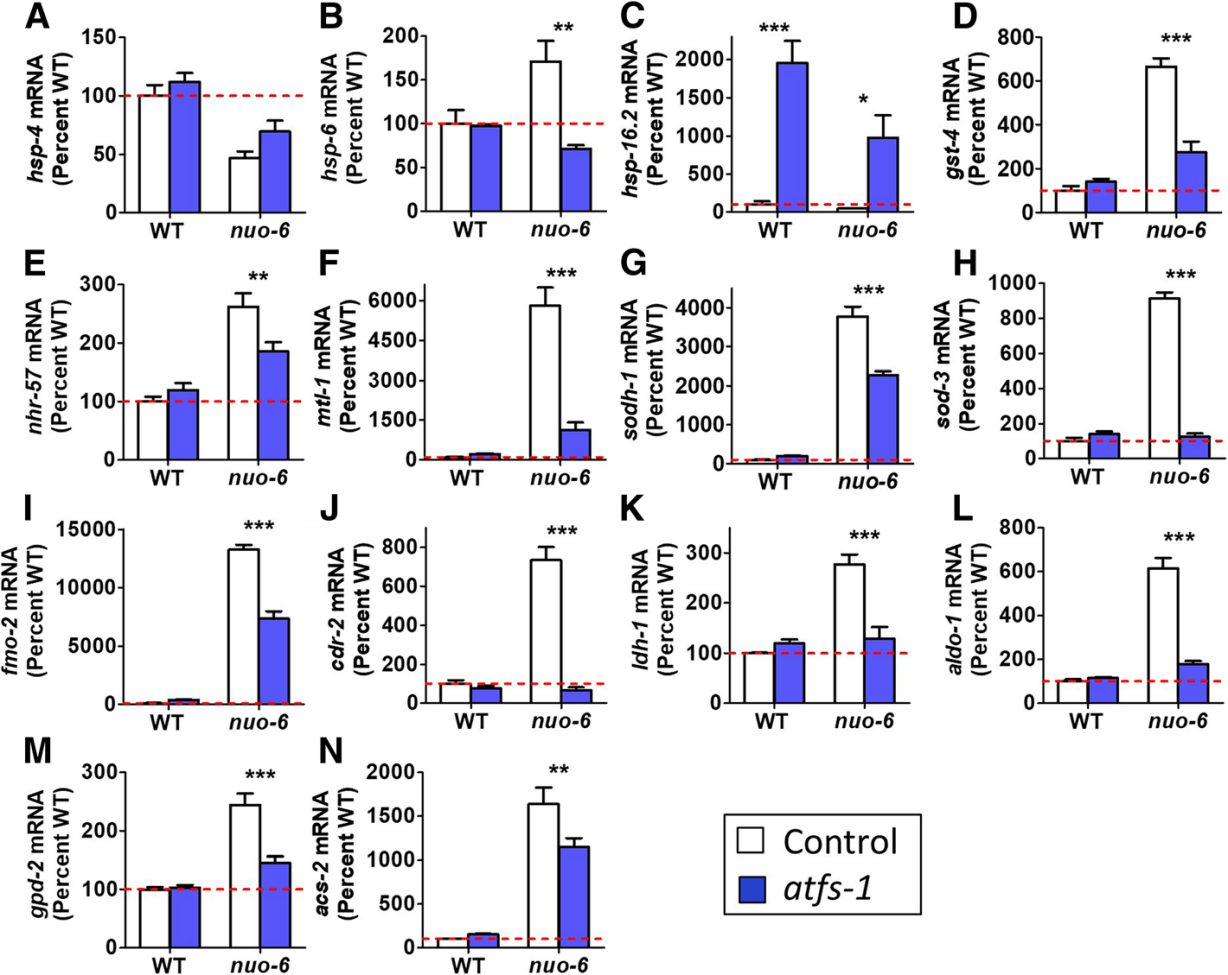
ATFS-1 mediates specific transcriptional changes in *nuo-6* worms. Quantitative real-time RT-PCR was used to compare the expression of selected stress response and metabolism genes between *nuo-6* and *nuo-6;atfs-1* worms. **a** *nuo-6* worms showed decreased expression of *hsp-4*, which is a marker of the endoplasmic reticulum unfolded protein response. **b** *hsp-6* expression was increased in *nuo-6* worms and this increase was completely prevented by loss of *atfs-1.* **c** Deletion of *atfs-1* resulted in increased expression of *hsp-16.2*, which is a marker of the cytosolic unfolded protein response. **d** *nuo-6* worms exhibited increased expression of the SKN-1/NRF2 target gene *gst-4*, which was prevented by *atfs-1* deletion. **e** Expression of the HIF-1 target gene *nhr-57* is increased in *nuo-6* worms and restored towards wild-type by the *atfs-1* deletion. The DAF-16/FOXO target genes *mtl-1* (**f**), *sodh-1* (**g**), and *sod-3* (**h**), are upregulated in *nuo-6* worms and require *atfs-1* for this upregulation. Similarly, *fmo-2* (**i**) and *cdr-2* (**j**), which are involved in xenobiotic detoxification, exhibit an ATFS-1-dependent increase in expression in *nuo-6* mutants. **k**–**n** Selected genes involved in metabolism (*ldh-1*, *aldo-1*, *gpd-2*, *acs-2*) show an ATFS-1-dependent increase in expression in *nuo-6* worms. Error bars indicate SEM. *p < 0.05,**p < 0.01,***p < 0.001. See Additional file 5: Table S5 for details on N and replicates

To further explore the mechanism by which the loss of *atfs-1* reduces the lifespan of *nuo-6* worms, we compared gene expression in *nuo-6* and *nuo-6;atfs-1* mutants. We hypothesized that ATFS-1-mediated changes in gene expression contribute to the long life of *nuo-6* worms. Accordingly, we examined the expression of genes involved in stress response or metabolism to identify genes that are modulated in *nuo-6* worms and restored towards wild-type by the loss of *atfs-1.* In contrast to the ATFS-1-dependent activation of the mitoUPR (*hsp-6*), neither the endoplasmic reticulum unfolded protein response (*hsp-4*) nor the cytosolic protein response (*hsp-16.2*) is activated in *nuo-6* worms (Fig. 6a–c).

Interestingly, deletion of *atfs-1* resulted in a marked activation of the cytosolic UPR as measured by increased *hsp-16.2* expression. Preliminary exploration of this result indicated that upregulation of *hsp-16.2* (and *hsp-16.11*) in *atfs-1* mutants is dependent on the heat shock factor HSF-1 but independent of the FOXO transcription factor DAF-16 (Additional file 1: Figure S12) [31]. To explore this relationship further, we generated *atfs-1;hsf-1* double mutants. We found that *atfs-1;hsf-1* double mutants are unhealthy as indicated by high sterility and low brood size. This suggests that the activation of the cytosolic UPR in *atfs-1* mutants may be a compensatory response for the disruption of the mitoUPR.

Examination of other stress response pathways revealed that the SKN-1-mediated stress response (*gst-4*), the HIF-1-mediated hypoxia response (*nhr-57*), and the DAF-16-mediated stress response (*mtl-1*, *sodh-1*, *sod-3*) are all activated in *nuo-6* worms (Fig. 6d–h; Additional file 1: Figure S13), which may contribute to the increased resistance to stress we observed in *nuo-6* mutants. In each case, loss of *atfs-1* reduced the extent to which each stress response was activated, suggesting that activation of the mitoUPR can lead to the activation of other stress response pathways. Note that we and others have also observed the upregulation of the HIF-1 target *nhr-57* in *clk-1* and *isp-1* mutants [27, 32, 33]. As ROS levels are elevated in *nuo-6* mutants [26], and elevated ROS is sufficient to activate the HIF-1 pathway [25], it is likely that ROS are responsible for HIF-1 activation in *nuo-6* worms.

We also examined the expression levels of *fmo-2*, a flavin-containing monoxygenase involved in xenobiotic detoxification that was recently shown to modulate longevity [34], and *cdr-2*, a glutathione S-transferase also involved in xenobiotic detoxification and implicated in the longevity of *isp-1* mutants [35]. As with the other stress response genes, we observed increased expression in *nuo-6* mutants that was significantly reduced by the loss of *atfs-1* (Fig. 6i, j). Note that other antioxidant enzymes, including superoxide dismutases (*sod-1*, *sod-2*, *sod-4*, *sod-5*), catalases (*ctl-1*, *ctl-2*, *ctl-3*), and peroxiredoxins (*prdx-2*, *prdx-6*), with the exception of *prdx-3*, were not affected by loss of *atfs-1* in wild-type or *nuo-6* worms (Additional file 1: Figure S14).

As *nuo-6* mutants have been shown to have altered expression of metabolic genes [36], we examined the role of ATFS-1 in the expression of select metabolic genes. We selected genes that we had previously found to be increased in *nuo-6* worms using RNAseq including lactate dehydrogenase (*ldh-1*), fructose biphosphate aldolase (*aldo-1*), glyceraldehyde 3-phosphate dehydrogenase (*gpd-2*), and fatty acyl-CoA synthetase (*acs-2*) [37]. We found that all of these genes showed increased expression in *nuo-6* worms that is dependent on *atfs-1* (Fig. 6k–n).

### Specific ATFS-1-regulated stress response and metabolism genes are required for *nuo-6* lifespan

To examine the role of specific changes in gene expression induced by the mitoUPR in the long lifespan of *nuo-6* mutants, we decreased the expression of genes that we found to be upregulated in *nuo-6* mutants in an ATFS-1-dependent manner and measured lifespan. We decreased the expression of *gst-4*, *mtl-1*, *sodh-1*, *fmo-2*, *cdr-2*, *ldh-1*, *aldo-1*, and *acs-2* using RNAi beginning at the L4 stage of the parental generation. For *hsp-6*, we begun RNAi treatment at the L4 stage of the experimental generation as *hsp-6* RNAi can induce embryonic lethality [38]. For *sod-3*, we used a genetic mutation rather than RNAi to avoid the possibility of knocking down its paralog *sod-2*, which could complicate the interpretation of the result since loss of *sod-2* increases lifespan [29]. We also used a genetic mutation for *hif-1* so that we could compare the results to previous findings in *clk-1* and *isp-1* mutants [33]. Note that conflicting results have been observed with respect to the longevity of the *hif-1* mutant [39, 40]. We used conditions in which the *hif-1* mutant is known to have an increased lifespan [41] such that any observed decrease in *nuo-6* lifespan resulting from *hif-1* mutation would be easier to interpret.

**Fig. 7:**
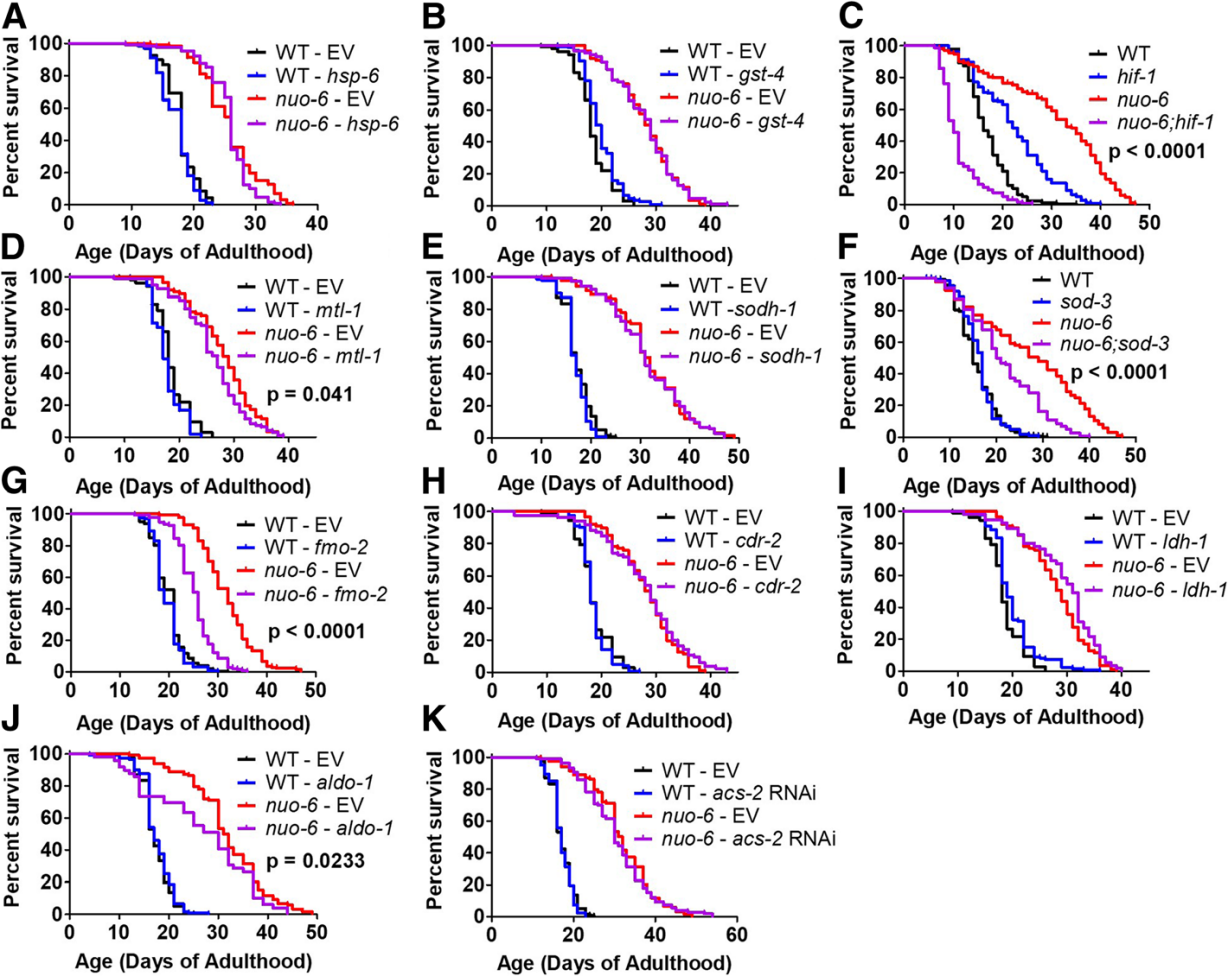
ATFS-1-regulated genes involved in stress resistance and metabolism are required for the long lifespan of *nuo-6* mutants. To explore the contribution of genes that show an ATFS-1-dependent upregulation in *nuo-6* mutants, we decreased the expression of these genes in *nuo-6* worms. We found that decreasing the expression of *hsp-6* (**a**), *gst-4* (**b**), *sodh-1* (**e**), *cdr-2* (**h**), *ldh-1* (**i**), or *acs-2* (**k**) using RNAi did not decrease the lifespan of *nuo-6* worms. In contrast, *hif-1* mutation (**c**), *mtl-1* RNAi (**d**), *sod-3* mutation (**f**), *fmo-2* RNAi (**g**), and *aldo-1* RNAi (**j**) significantly reduced *nuo-6* longevity. This indicates that changes in gene expression induced by the activation of the mitoUPR contribute to the long-lifespan of *nuo-6* worms. Significance between red and purple lines is indicated. See Additional file 4: Table S4 for raw lifespan data. See Additional file 5: Table S5 for details on N and replicates

We found that decreasing the expression of *hsp-6*, *gst-4*, *sodh-1*, *cdr-2*, and *ldh-1* did not affect the survival of *nuo-6* or wild-type worms (Fig. 7a, b, e, h, i). In contrast, we found that knockdown of *mtl-1*, *fmo-2*, or *aldo-1* all significantly reduced *nuo-6* longevity but had no effect on wild-type lifespan (Fig. 7d, g, j). Similarly, *nuo-6;sod-3* and *nuo-6;hif-1* double mutants exhibited decreased lifespan compared to *nuo-6* mutants (Fig. 7c, f). Combined, this demonstrates that changes in the expression of stress response genes (*hif-1*, *mtl-1*, *fmo-2*, *sod-3*) and genes involved in metabolism (*aldo-1*) that are induced by ATFS-1 contribute to the longevity of *nuo-6* mitochondrial mutants.

Because the mutation in *hif-1* completely abolished the lifespan increase present in *nuo-6* worms such that *nuo-6;hif-1* double mutants live shorter than wild-type worms (Fig. 7c), we decided to further explore the role of *hif-1* in *nuo-6* longevity. To do this, we used RNAseq to compare gene expression in *nuo-6* and *nuo-6;hif-1* mutants in order to identify genes that are modulated in *nuo-6* worms and restored to wild-type by the *hif-1* mutation, as these genes may contribute to the long life of *nuo-6* worms. Using this approach, we found numerous upregulated or downregulated genes in *nuo-6* worms that are HIF-1 dependent (Additional file 1: Figure S15). While it is beyond the scope of our current study to explore these targets in depth, our initial enrichment analyses identified genes involved in various types of metabolism as the top categories of upregulated genes that are reverted by the *hif-1* mutation, while transport/trafficking genes are enriched among the downregulated genes (Additional file 1: Figures S16, S17).

### ATFS-1 affects the expression of DAF-16 target genes

Because we observed an ATFS-1-dependent activation of three DAF-16 target genes in *nuo-6* mutants, we decided to further explore the role of ATFS-1 in the activation of DAF-16 target genes. To do this, we examined the effect of the *atfs-1* deletion on the nuclear localization of DAF-16 using a DAF-16 translational reporter strain (*zIs356[Pdaf-16::daf-16:GFP]*) [42]. We found that in *atfs-1* deletion mutants, the nuclear localization of DAF-16 in response to 35 °C heat stress was significantly slowed (Additional file 1: Figure S18), suggesting that ATFS-1 can facilitate nuclear localization of DAF-16.

Since loss of ATFS-1 affected the nuclear localization of DAF-16, we next wondered the extent to which activation of ATFS-1 caused modulation of DAF-16 target genes. We had previously shown that the transcriptional changes present in *nuo-6* mutants exhibit a significant overlap with *daf-2* mutants and a significant enrichment of DAF-16 target genes and that DAF-16 is required for the long lifespan of *nuo-6* mutants [37]. To more directly assess the role of ATFS-1 in modulation of DAF-16 target genes, we used RNAseq to examine gene expression in two *atfs-1* gain-of-function mutants. These two mutant, *atfs-1(et15)* and *atfs-1(et17)*, have point mutations affecting the mitochondrial targeting sequence, which results in cytoplasmic accumulation of ATFS-1 allowing ATFS-1 to enter the nucleus and activate its transcriptional targets [43].

**Fig. 8:**
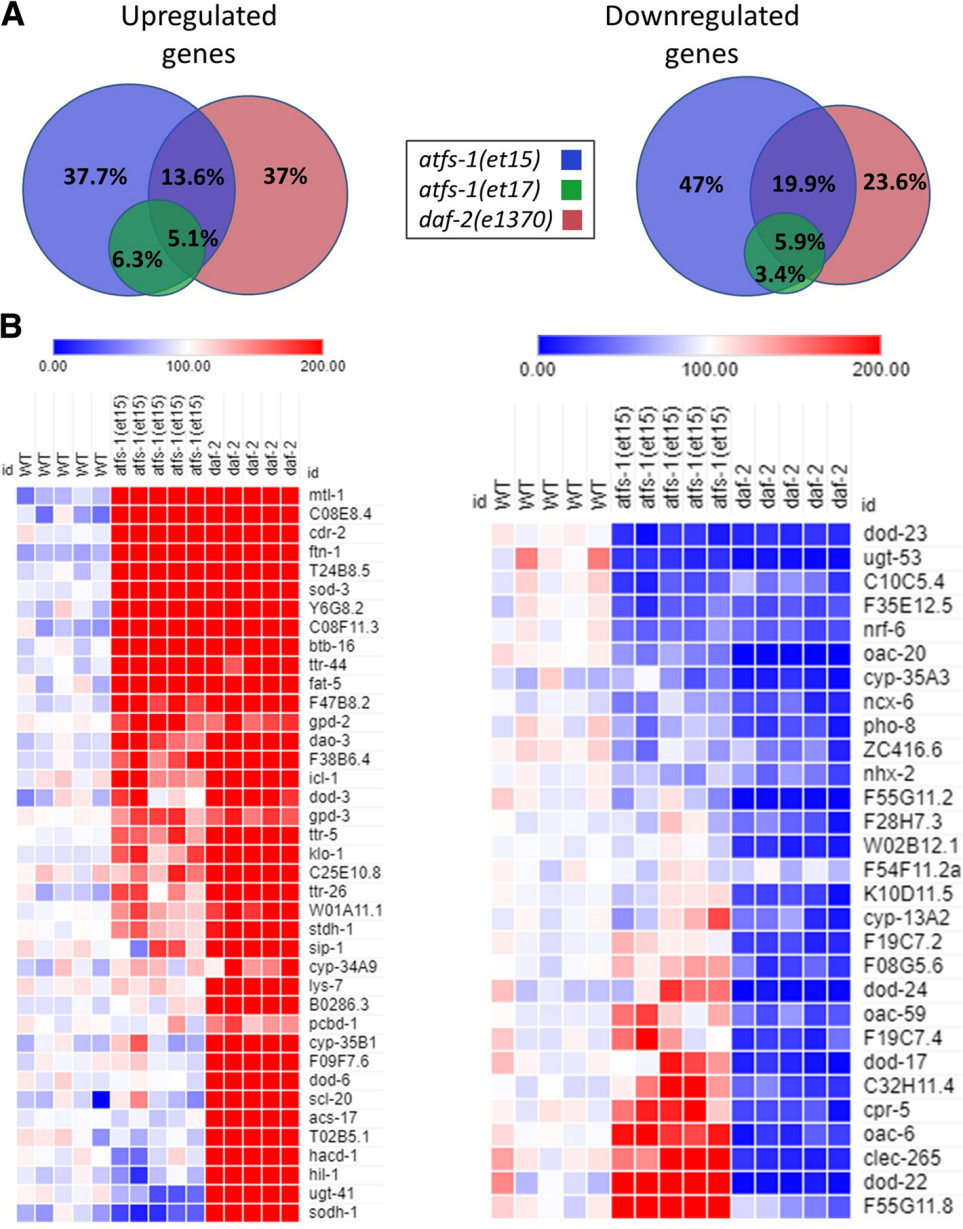
Gene expression changes in gain-of-function *atfs-1* mutants exhibit significant overlap with *daf-2* mutants and enrichment of DAF-16 target genes. **a** Venn diagrams indicate the overlap in differentially expressed genes between the two *atfs-1* gain-of-function mutants (*et15* and *et17*) and *daf-2(e1370).* The changes in gene expression in *atfs-1*(*et17)* almost completely overlap with those in *atfs-1(et-15).* Percentages indicate the percent of all of the genes that are differentially expressed in one of the three mutants. **b** Heat maps showing the expression of DAF-16 target genes in *atfs-1* gain-of-function mutants compared to *daf-2.* The genes shown are the top DAF-16 target genes determined by a meta-analysis of gene expression data performed by Tepper et al., 2013. Only genes that showed a significant change in expression in *daf-2* mutants are shown. The *atfs-1* gain-of-function mutants show upregulation of many of the top DAF-16 upregulated genes and downregulation of many of the top DAF-16 downregulated genes. However, some genes show the opposite pattern of regulation

We found that the upregulated and downregulated genes in the *atfs-1* gain-of-function mutants exhibited a significant degree of overlap with *daf-2(e1370)* mutants (Fig. 8a, Additional file 1: Table S1). In addition, many of the top upregulated and downregulated DAF-16 target genes determined by a meta-analysis of *daf-16* expression data [44] were similarly modulated in the *atfs-1* gain-of-function mutants (Fig. 8b). While further studies will be required to explore the molecular mechanisms, these results suggest that ATFS-1 can affect DAF-16 target genes at least partially through the nuclear localization of DAF-16.

### ATFS-1 dependent transcriptional changes in *nuo-6* mutants

Because we identified multiple ATFS-1-dependent changes in gene expression in *nuo-6* worms that contributed to longevity, we decided to use RNAseq to gain a more comprehensive view of which transcriptional changes in *nuo-6* mutants are ATFS-1 dependent (see Additional file 2: Table S2 for differentially expressed genes). We identified many genes that were upregulated or downregulated in *nuo-6* worms and restored to wild-type by the *atfs-1* deletion (Additional file 1: Figure S19). While further studies will be required to assess the contribution of these genes to *nuo-6* longevity, our initial enrichment analysis shows that genes involved in metabolism of lipids are the most enriched upregulated gene class that is dependent on ATFS-1, while genes involved in the metabolism of RNA are the most enriched downregulated gene class that is ATFS-1 dependent (Additional file 1: Figures S20, S21). Congruent with our results showing a role for ATFS-1 is stress resistance, resistance to stress was one of the GO terms identified for ATFS-1-dependent upregulated genes in *nuo-6* mutants (Additional file 3: Table S3).

**Fig. 9:**
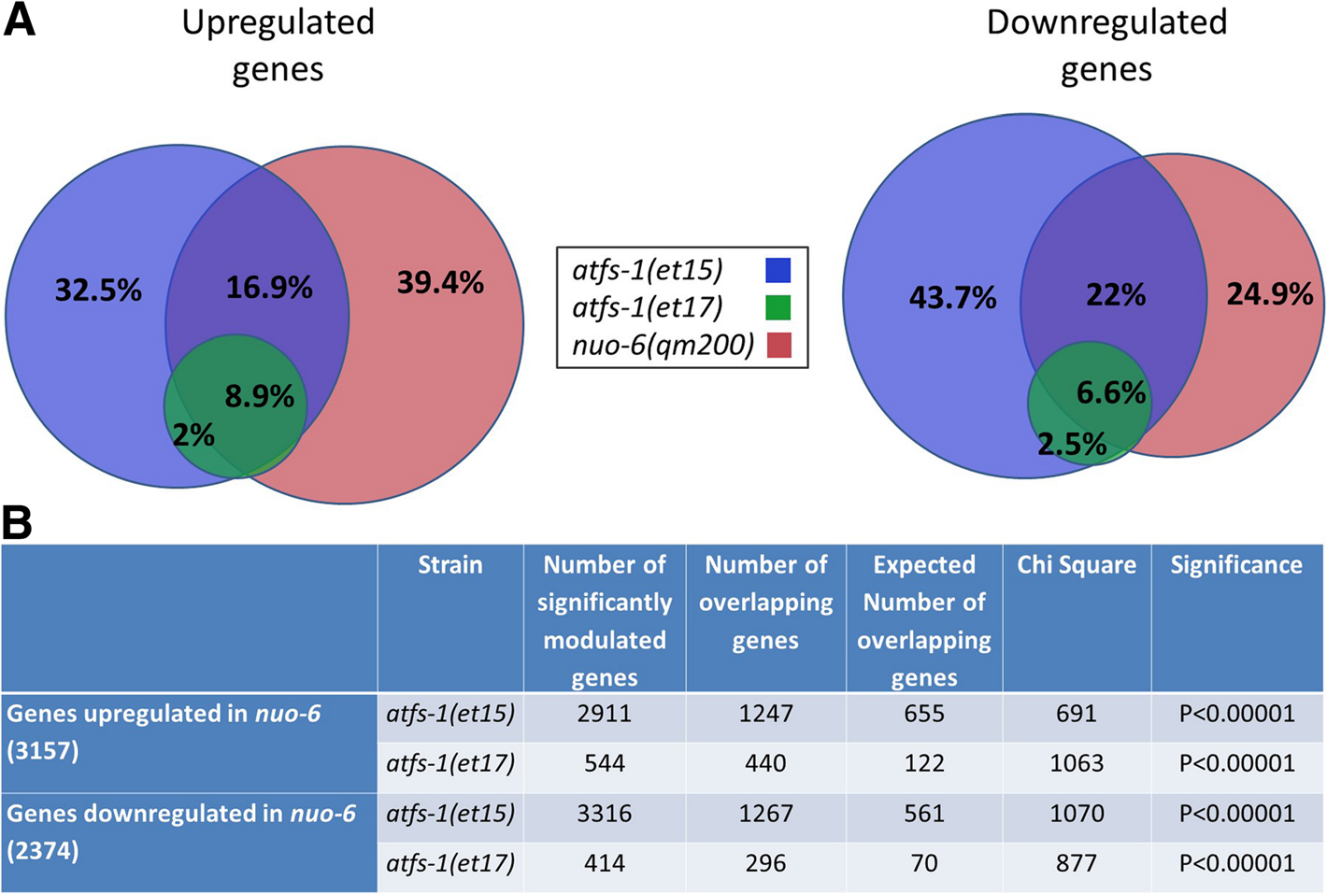
*atfs-1* gain-of-function mutants show a highly significant overlap in gene expression changes with *nuo-6* mutants. **a** Venn diagrams indicate the overlap in differentially expressed genes between the two *atfs-1* gain-of-function mutants (*et15* and *et17*) and *nuo-6(qm200)* worms*.* Percentages indicate the percent of all of the genes that are differentially expressed in one of the three mutants. **b** For both upregulated genes and downregulated genes, the degree of overlap is highly significant

Having shown that *nuo-6* worms exhibit activation of the mitoUPR and a large number of ATFS-1-dependent changes in gene expression, we compared the gene expression of *nuo-6* mutants to the *atfs-1* gain-of-function mutants, *atfs-1(et15)* and *atfs-1(et17)*. We found that there is a highly significant degree of overlap between *nuo-6* mutants and the *atfs-1* gain-of-function mutants (Fig. 9). We also observed a highly significant degree of overlap between both *clk-1* mutants and *isp-1* mutants and the *atfs-1* gain-of-function mutants (Additional file 1: Figures S22, S23). Combined, this suggests that a large proportion of transcriptional changes in the long-lived mitochondrial mutants are caused by activation of ATFS-1.

## Discussion

### Role of mitochondrial unfolded protein response in development and lifespan of long-lived mitochondrial mutants

The mitochondrial gene mutations *clk-1*, *isp-1*, and *nuo-6* affect the function of the electron transport chain, leading to increased longevity [19–22]. Since the activation of the mitoUPR improves protein homeostasis in the mitochondria, it is plausible that this activation could improve longevity. While it has been clearly demonstrated that activation of the mitoUPR is not sufficient to increase lifespan [16], contribution of the mitoUPR to the longevity of specific long-lived mutants or RNAi-treated worms has been debated [12, 16]. In our current study, we provide resolution to these differing results by using multiple paradigms, exploring the role of the mitoUPR at different developmental stages, and measuring the precise levels of knockdown for each condition.

By knocking down *atfs-1* levels specifically during adulthood, we show that ATFS-1 is not required during adulthood for the longevity of *clk-1*, *isp-1*, or *nuo-6* worms. Assessing the role of ATFS-1 and the mitoUPR during development in the longevity of the long-lived mitochondrial mutants is more complicated and has likely lead to the differing views of previous publications. Our results suggest that if *atfs-1* levels are knocked down sufficiently, it is not possible to fully assess the role of ATFS-1 in the lifespan of *clk-1* and *isp-1* worms because *atfs-1* is required for these worms to develop to adulthood (*atfs-1* RNAi beginning in the parental generation or *atfs-1* deletion both resulted in developmental arrest). Thus, when testing the role of ATFS-1 in longevity, our results suggest that it is essential to quantify the extent of knockdown using RNAi or to use a null mutant.

The fact that *nuo-6;atfs-1* mutants are able to develop to adulthood provided us with the opportunity to assess the role of *atfs-1* in the longevity of a long-lived mitochondrial mutant. We found that a deletion of *atfs-1* reverts the lifespan of *nuo-6* worms back to wild-type lifespan, while *atfs-1* RNAi beginning in the parental generation markedly decreases *nuo-6* lifespan. Combined, our results demonstrate that *atfs-1* performs an essential role during development which allows *clk-1* and *isp-1* worms to reach adulthood; however, once these worms reach adulthood, *atfs-1* is expendable with respect to longevity. Unlike *clk-1* and *isp-1* mutants, *nuo-6* worms are able to tolerate the absence of *atfs-1* during development, but *atfs-1* is required for their long lifespan.

### Mitochondrial unfolded protein response is required for resistance to multiple forms of stress

The mitoUPR is activated by stresses that disrupt protein folding or stoichiometry in the mitochondria [2, 5, 6]. While a role for ATFS-1 was previously demonstrated in resistance to bacterial pathogens and anoxia [45, 46], our work demonstrates that the mitoUPR has a much broader impact on resistance to multiple forms of stress. Despite the fact that we have previously shown that the mitoUPR, as measured using the *Phsp-6::GFP* reporter strain [2], is activated by oxidative stress but not heat, cold, osmotic, anoxic, starvation, ER or bacterial pathogen stress [25], the loss of the mitoUPR results in increased sensitivity to multiple stresses. Deletion of *atfs-1* increased sensitivity to oxidative, osmotic, heat, and anoxic stress in both wild-type and *nuo-6* worms. The mechanism by which ATFS-1 modulates stress resistance is likely through the upregulation of stress response pathways, as we show that deletion of *atfs-1* prevents the upregulation of target genes of the HIF-1-mediated hypoxia response (*nhr-57*), the SKN-1-mediated oxidative stress response (*gst-4*), the DAF-16-mediated stress response (*mtl-1*, *sodh-1*, *sod-3*), and genes involved in xenobiotic detoxification (*fmo-2*, *cdr-2*) in *nuo-6* worms. Combined, our results suggest that activation of the mitoUPR causes an ATFS-1-dependent increase in the expression of stress response genes from multiple pathways leading to an increased stress resistance.

### Loss of mitochondrial unfolded protein response causes activation of cytosolic unfolded protein response

In examining the effect of *atfs-1* deletion on the expression of genes involved in known stress response pathways, we observed a dramatic upregulation of *hsp-16.11* and *hsp-16.2*, two genes involved in the cytosolic unfolded protein response (cytoUPR), but no effect on the expression of *hsp-4*, which is involved in the ER unfolded protein response (ER-UPR). We also found that knocking down other proteins involved in the mitoUPR, including *ubl-5* and *dve-1*, resulted in upregulation of the cytosolic unfolded protein response target genes. Similarly, *hsp-16.2* was also found to be upregulated when the *atfs-1* target gene *hsp-6* was knocked down through a mechanism involving lipid biosynthesis [47]. In contrast, we found that knockdown of *haf-1* did not affect *hsp-16.11* or *hsp-16.2* expression. Although heat stress has been shown to activate the unfolded protein response in multiple compartments of the cell, many stresses activate only one of the mitoUPR, cytoUPR, or ER-UPR [25]. Consistent with this observation, *nuo-6* mutants, which exhibit activation of the mitoUPR (as measured by *hsp-6* reporter and expression), show decreased expression of genes involved in the cytoUPR (*hsp-16.2*) and ER-UPR (*hsp-4*). The fact that the cytoUPR is activated when *atfs-1* is knocked down suggests that there may be communication between the mitoUPR and cytoUPR to protect the cytoplasm from potential detrimental effects of a defective mitoUPR. This conclusion is supported by our data showing that blocking the cytoUPR in mitoUPR-deficient *atfs-1* mutants leads to a general decline in worm health. It is also supported by work showing that decreasing the levels of a mitochondrial protein, cytochrome c oxidase (F29C4.2), causes both an ATFS-1-dependent activation of the mitoUPR and delays the age-associated decline of the cytoUPR in an HSF-1-dependent manner [17]. Combined, these results suggest that the mitoUPR and cytoUPR work together to maintain proteostasis in the cell.

### Longevity of *nuo-6* worms is mediated by ATFS-1-mediated changes in the expression of stress response and metabolism genes

In this work, we show that *nuo-6* worms exhibit increased resistance to multiple forms of stress, and upregulation of genes involved in multiple stress response pathways including the SKN-1-mediated oxidative stress response (*gst-4*), HIF-1-mediated hypoxia response (*nhr-57*), DAF-16-mediated stress response (*mtl-1*, *sodh-1*, *sod-3*), and xenobiotic detoxification genes (*fmo-2*, *cdr-2*). The upregulation of all of these stress response genes requires *atfs-1* suggesting that the mechanism by which *atfs-1* promotes longevity in *nuo-6* worms may be at least partially mediated by increasing resistance to stress. Accordingly, we assessed the contribution of individual ATFS-1-modulated genes or pathways to *nuo-6* lifespan. The loss of the hypoxia inducible factor *hif-1* had the greatest impact on *nuo-6* lifespan as *nuo-6;hif-1* double mutants live shorter than wild-type worms. As it has previously been reported that *hif-1* is required for the longevity of *clk-1* and *isp-1* mutants [33], and multiple genes involved in the HIF-1-mediated hypoxia response have been shown to be upregulated in *isp-1* worms [27], this suggests that the hypoxia response pathway may be a common mediator of longevity among multiple mitochondrial mutants.

The second largest impact on *nuo-6* lifespan that we observed resulted from the knockdown of the Flavin-containing monooxygenase *fmo-2.* This gene has also been shown to be required for the increased lifespan of *vhl-1* mutants, which have constitutive activation of the hypoxia response, and the long lifespan resulting from dietary restriction on solid plates [34]. Combined, this suggests that *fmo-2* may be a common downstream effector of multiple pathways of lifespan extension.

The loss of *sod-3*, the inducible mitochondrial *sod* gene, also markedly decreased *nuo-6* lifespan, despite the fact that *sod-3* expression normally accounts for less than 1% of total *sod* mRNA, and accounts for less than 8% of *sod* mRNA in *nuo-6* worms. We have previously shown that *sod-3* is also required for the long lifespan of *isp-1* mutants [27], but is expendable for *clk-1* and wild-type lifespan [28, 29, 32]. The increased dependence of *nuo-6* and *isp-1* worms on *sod-3* expression may stem from the fact that *clk-1* worms show a more widespread upregulation of antioxidant defense genes [32], than *isp-1* [27] or *nuo-6* mutants.

## Conclusions

The ATFS-1 transcription factor, which mediates the mitoUPR, is required for the development and lifespan of the long-lived mitochondrial mutants, but is dispensable during adulthood. Our results demonstrate a novel role for ATFS-1 in resistance to stress and suggest that the mechanism by which ATFS-1 modulates longevity in *nuo-6* worms is through the activation stress response pathways.

## Methods

### Strains

Strains were maintained on nematode growth medium (NGM) plates at 20 °C with OP50 *E. coli* bacteria as the food source. All experiments were performed using live bacteria. The following strains were utilized in this study: wild-type (N2 Bristol), *clk-1(qm30)*, *isp-1(qm150)*, *nuo-6(qm200)*, *sod-2(ok1030)*, *sod-3(tm760)*, *fmo-2(ok2147)*, *atfs-1(gk3094)*, *atfs-1(et15)*, *atfs-1(et17)*, *hsf-1(sy441)*, *daf-16(mu86)*, *hif-1(ia4)*, *zcIs13[Phsp-6::GFP]*, *dvIs19[Pgst-4::GFP]*, *iaIs7[Pnhr-57::GFP]*, *muIs84[Psod-3::GFP]*, *zIs356[Pdaf-16::daf-16:GFP]*, *nuo-6(qm200);atfs-1(gk3094)*, *clk-1(qm30);zcIs13[Phsp-6::GFP]*, *isp-1(qm150);zcIs13[Phsp-6::GFP]*, *nuo-6(qm200);zcIs13[Phsp-6::GFP]*, *nuo-6(qm200);dvIs19[Pgst-4::GFP]*, *nuo-6(qm200);muIs84[Psod-3::GFP]*, *nuo-6(qm200);sod-3(tm760)*, *nuo-6(qm200);fmo-2(ok2147)*, *nuo-6(qm200);hif-1(ia4)*, *atfs-1(gk3094);hsf-1(sy441)*, *atfs-1(gk3094);daf-16(mu86)*, *atfs-1(gk3094); zIs356[Pdaf-16::daf-16:GFP].* Double mutants were generated as described [48] and confirmed in two consecutive generations. *atfs-1(gk3094)* is an 881 base pair deletion beginning in exon 3 that is predicted to result in a frameshift that would affect the leucine zipper domain and nuclear localization signal at the C-terminus of the protein. Thus, it is likely that *gk3094* is a null allele.

### Quantification of fluorescence in reporter strains

Adult worms were immobilized using 20 mM sodium azide, arranged into a group of 8 worms and imaged using VimbaViewer 1.1.2 software and an AVT Stingray F145B camera connected to a Nikon SMZ1500 microscope. Quantification of fluorescence was performed using ImageJ software to measure integrated density in whole individual worms. Three replicates of 8 worms each were examined. Quantification of fluorescent reporter strains during larval development was performed in liquid in a 96-well dish using a Cellomics Arrayscan high content imager [32]. Biological replicates were performed on different days from distinct populations of worms.

### Nuclear localization of DAF-16

Nuclear localization of DAF-16 was visualized using a reporter strain in which *daf-16* is fused to GFP: TJ356 *zIs356 [Pdaf-16::daf-16a/b:GFP+rol-6(su1006)]* [42]. Worms were exposed to a mild heat stress at 35 °C for 30 min on 60 mm NGM plates and then immobilized using levamisole on an unseeded NGM plate prior to visualization using a Nikon SMZ1500 fluorescence dissecting microscope.

### RNAi

RNAi clones picked from the Ahringer RNAi library were sequenced to confirm that they target the correct gene. RNAi plates containing 50 μM carbenicillin and 3 mM IPTG were seeded with 150 μL of 5× concentrated RNAi bacteria that were grown for less than 12 h in LB media containing 50 μM carbenicillin. Plates were allowed to sit for 2 days to allow for bacterial growth and induction of RNAi construct before transferring worms onto the plates. Plates were made fresh weekly and plates that were not used immediately were stored in a 4 °C cold room. Two different RNAi paradigms were utilized to avoid detrimental effects of gene knockdown during development. In the L4 parental paradigm, worms are transferred to RNAi plates at the L4 stage of development. When the worms become gravid adults, they are transferred to a second RNAi plate and allowed to lay eggs for 1 day. Experiments are then performed on the F1 progeny. In the L4 direct paradigm, the experimental animals are transferred to RNAi plates at the L4 stage of development and experiments are performed directly on these animals.

### Lifespan assays

Lifespan assays were performed at 20 °C on plates containing FUdR. In most cases, we used 25 μM FUdR as this concentration was shown to have minimal effects on longevity while effectively preventing the development of progeny on the second lifespan plate [49]. For experiments involving *clk-1*, we used 100 μM FUdR as these worms tend to have a high rate of non-aging related pathologies at lower concentrations of FUdR. Lifespan was performed in a blinded manner by three independent researchers. Worms were transferred to fresh plates weekly. Worms were removed from the experiment if they died of unnatural causes including desiccation on the side of the dish, internal hatching of progeny (bagging) or expulsion of internal organs. Lifespan data is provided in Additional file 4: Table S4.

### Measurement of mRNA levels

RNA was isolated from synchronized populations on pre-fertile young adult worms using TRIZOL as previously described [50]. RNA was isolated from a plate of synchronized worms containing approximately 1000 worms. For quantitative real-time RT-PCR, mRNA was converted to cDNA using a High Capacity cDNA Reverse Transcription Kit (Life Technologies) according to the manufacturer’s protocol. qPCR was performed in an AP Biosystems RT-PCR machine using a FastStart Universal SYBR Green kit (Roche). mRNA levels from at least three biological replicates collected on different days were normalized to the levels of *act-3* and then expressed as a percentage of wild-type*.*

### RNA sequencing and analysis

RNA sequencing (RNAseq) data will be publicly available at GEO: GSE110984. RNA sequencing and analysis was performed as described previously [27]. For RNAseq experiments, we collected at least three independent samples for all genotypes. RNA was isolated independently for all samples. Sequencing libraries were prepared from 500 ng of total RNA using the Kapa Biosystems stranded mRNA-seq kit for the Illumina platform. Libraries were pooled equimolarly and sequenced to a minimum depth of 30 M reads using 1 × 75 bp sequencing on the Illumina NextSeq 500 platform at the Van Andel Research Institute. Read quality was assessed using FastQC v. 0.11.5 (http://www.bioinformatics.babraham.ac.uk/projects/fastqc/) and one-pass aligned to WBcel235 *C. elegans* genome using STAR v. 2.5.2b [51] with default parameters and “--outReadsUnmapped None”. The STAR genome index was generated with the corresponding Ensembl WBcel235 build 89 GTF annotations integrated. Transcript abundances were quantified using the --quantMode GeneCounts option enabled during alignment. Differential gene expression analysis was performed using the quasi-likelihood framework in edgeR v. 3.20.2 [52] in R v. 3.4.1, adjusting for sequencing batch in the model. Gene names, gene IDs, and predicted function annotations were downloaded using the biomaRt package v. 2.34.2. Gene ontology enrichment was performed with the GOseq package v. 1.30.0 [53]. Reactome enrichment was performed using the ReactomePA package v. 1.22.0 reactome [54]. Venn diagrams of overlapping genes were generated using the free online tool BioVenn: http://www.biovenn.nl/. Heat maps were generated using the free online tool from the Broad Institute Morpheus: (https://software.broadinstitute.org/morpheus/).

### Quantification of physiologic rates

Embryonic lethality was measured by allowing worms to lay eggs for a period of 2–4 h. After 2 days, the number of unhatched eggs and live worms were counted to determine embryonic lethality. Post-embryonic development time was measured by picking 100–300 eggs to a new plate. After 3 h, 25 newly hatched L1 worms were transferred to a new plate. Development to adulthood was checked every 4 h beginning at 36 h. Worms that failed to develop to adulthood during the post-embryonic development time assay were recorded to quantify developmental arrest. Brood size was determined by transferring L4 worms to individual plates. Worms were then transferred daily when progeny production began. When progeny grew to adulthood, plates were stored at 4 °C to immobilize worms prior to counting offspring. Thrashing rate was manually counted by transferring 20 pre-fertile young adult worms to an NGM plate without bacteria. One milliliter of M9 buffer was then added to the plate and the number of body bends per 30 s was counted. Defecation cycle length was counted as the time between two consecutive pBoc contractions. Worms were plated on NGM plates completely covered in bacteria at the L4 stage of development and defecation was measured the following day. Body length was determined by anesthetized worms with 10 mM sodium azide, imaging worms and using ImageJ to determine length compared to a calibration ruler.

### Mitochondrial function

Oxygen consumption rate was measured using a Seahorse XFe96 analyzer (Seahorse bioscience Inc., North Billerica, MA, USA) [55]. Day 1 adult worms were washed in M9 buffer and transferred into a Seahorse 96-well plate containing 175 μL Seahorse calibrant. Oxygen consumption was measured in five biological replicates, each of which included 5 technical replicates, all at room temperature. Each replicate included approximately 20 worms. Oxygen consumption was normalized per worm rather than per milligram protein. By using the extracellular flux analyzer, we were able to quantify the exact numbers of worms present and to perform more replicates than would be possible by traditional Clarke electrode approaches. However, in order to prevent overcrowding, the number of worms utilized per well in the extracellular flux analyzer is too low to get an accurate measurement of protein.

To measure ATP levels, approximately 200 worms were age-synchronized by transferring daily. Worms were collected in de-ionized water, washed, and freeze-thawed three times. The resulting pellet was sonicated in a Bioruptor (Diagenode) with 30 cycles of 30 s on and 30 s off. The pellet was boiled for 15 min to release ATP, then spun at 4° at 11,000*g* for 10 min. The supernatant was collected and measured using a Molecular Probes ATP determination Kit (Life Technologies). Luminescence was normalized to protein content, which was measured with a Pierce BCA protein determination kit (Thermo Scientific). Statistical analysis was performed using GraphPad software.

To assess mitochondrial morphology, worms were crossed to *Pmyo-2::mito-GFP* worms and age-synchronized by transferring daily. For imaging, worms were transferred to an agar pad mounted on a slide. Worms were subsequently paralyzed with 10 μM levamisole and cover-slipped. Images were captured by confocal microscopy (Nikon A1R Ti) at 60×. Images were processed and analyzed using Nikon Elements Basic Research software. Background was subtracted and GFP was thresholded at a constant for all images. We found that the *atfs-1* mutation inhibited the correct localization of GFP to the mitochondria [55], and thus we were unable to examine mitochondrial morphology using this approach.

To measure the levels of ROS, we used the ROS-sensitive dye dichlorofluorescein (DCF) based on a published protocol [56]. Fifty L1 worms per well were incubated in 25 μM DCF for 2 h. Each strain was measured six times. As a positive control, worms were treated with 5 mM of the ROS-generating compound paraquat. Fluorescence was measured every 10 min for an hour using a plate reader.

### Sensitivity to stress

Sensitivity to heat stress was assessed by transferring worms to 37 °C and monitoring survival hourly. Sensitivity to oxidative stress was measured by transferring worms to plates containing 2 mM paraquat. These plates also contained 100 μM FUdR to prevent internal hatching of progeny, which can be caused by treatment with paraquat. Sensitivity to osmotic stress was assessed by transferring worms to NGM plates containing 550 mM NaCl and testing survival after 2 days. Sensitivity to anoxia was assessed using a BD Bio-Bag Type A environmental chamber (Becton, Dikinson and Company, NJ) according the manufacturer’s directions. Worms were placed under anoxic conditions for 54 h and allowed 24 h under normoxia to recover before scoring survival. Three replicates of at least 20 worms per replicate were performed.

### Statistical analysis

Statistical analysis was performed using GraphPad Prism version 5.01. Statistical significance of survival data was determined using the log-rank test. Experiments involving a time course were analyzed using a repeated measures ANOVA. The remaining experiments were analyzed using one-way or two-way ANOVA depending on the number of groups and outcomes. To determine which genes from the RNAseq data are significantly modulated, we used the quasi-likelihood framework in edgeR package v. 3.20.1 [52] in R v. 3.4.1. To assess the significance of overlapping genes between two strains, we used Chi square tests to determine if the number of overlapping upregulated or downregulated genes between the two strains was significantly greater than what would be expected by chance. Error bars indicate standard error of the mean. *p < 0.05, **p < 0.01, ***p < 0.001. For additional details on N for each experiment, please see Additional file 5: Table S5.

## Additional files

Additional file 1: **Figure S1.** *hsp-6* mRNA levels are increased in young adult *clk-1*, *isp-1* and *nuo-6* worms and correlated with *atfs-1* mRNA levels. **Figure S2.** Activation of the mitochondrial unfolded protein response in long-lived mitochondrial mutants is dependent on *atfs-1.* **Figure S3.** Treatment with antioxidants does not prevent activation of the mitochondrial unfolded protein response in *nuo-6* mutants. **Figure S5.** ATFS-1 is required for development to adulthood in *clk-1* and *isp-1* worms. **Figure S6.** *nuo-6* worms requires UBL-5 and DVE-1 to develop to adulthood*.* **Figure S7.** *haf-1* is not required for the longevity of the long-lived mitochondrial mutant *nuo-6*. **Figure S8.** Effect of knocking down components of the mitochondrial unfolded protein response on activation of the cytoplasmic unfolded protein response. **Figure S9.** Mitochondrial unfolded protein response is activated in *sod-2* mutants but not required for their longevity. **Figure S10.** Restoring *atfs-1* expression during adulthood reverts lifespan to *nuo-6.* **Figure S11.** Disruption of *atfs-1* decreases levels of reactive oxygen species (ROS). **Figure S12.** Activation of cytosolic unfolded protein response in *atfs-1* mutants is HSF-1-dependent and DAF-16-independent. **Figure S13.** ATFS-1 is required for the activation of stress response pathways in *nuo-6* worms. **Figure S14.** The expression of antioxidant defense genes is not significantly modulated by *atfs-1* deletion. **Figure S15.** HIF-1-regulated gene expression changes in *nuo-6* mitochondrial mutant. **Figure S16.** Reactome enrichment for genes upregulated in *nuo-6* mutants and reverted to wild-type in *nuo-6;hif-1* double mutants. **Figure S17.** Reactome enrichment for genes downregulated altered in *nuo-6* mutants and reverted to wild-type in *nuo-6;hif-1* double mutants. **Figure S18.** Loss of *atfs-1* decreases nuclear localization of DAF-16. **Figure S19.** ATFS-1-regulated gene expression changes in *nuo-6* mitochondrial mutant. **Figure S20.** Reactome enrichment for genes upregulated in *nuo-6* mutants and reverted to wild-type in *nuo-6;atfs-1* double mutants. **Figure S21.** Reactome enrichment for genes downregulated in *nuo-6* mutants and reverted to wild-type in *nuo-6;atfs-1* double mutants. **Figure S22**. *atfs-1* gain-of-function mutants show a highly significant overlap in gene expression changes with *clk-1* mutants. **Figure 23**. *atfs‐1* gain‐of‐function mutants show a highly significant overlap in gene expression changes with *isp‐1* mutants. **Table S1**. *atfs‐1* gain‐of‐function mutants exhibit statistically significant overlap in gene expression  changes with* daf‐2* mutants. (PDF 2446 kb)

Additional file 2: **Table S2.** Lists of significantly modulated genes in RNAseq studies. (XLSX 2726 kb)

Additional file 3: **Table S3.** GO term enrichment for genes upregulated and downregulated in *nuo-6* mutants and restored to wild-type in *nuo-6;atfs-1* mutants. (XLSX 17 kb)

Additional file 4: **Table S4.** Raw data from lifespan studies. (XLSX 47 kb)

Additional file 5: **Table S5.** Information on minimum reporting standards. (XLSX 12 kb)

## Abbreviations

ANOVA: Analysis of variance
ATP: Adenosine triphosphate
cDNA: Complementary deoxyribose nucleic acid
cytoUPR: Cytoplasmic unfolded protein response
DCF: Dichlorofluorescein
ER-UPR: Endoplasmic reticulum unfolded protein response
EV: Empty vector
FUdR: 5-Fluoro-2′-deoxyuridine
IPTG: Isopropyl β-D-1-thiogalactopyranoside
mitoUPR: Mitochondrial unfolded protein response
mRNA: Messenger RNA
NGM: Nematode growth media
PCR: Polymerase chain reaction
RNA: Ribonucleic acid
RNAi: RNA interference
RNAseq: RNA sequencing
ROS: Reactive oxygen species
RT-PCR: Reverse transcriptase PCR
