# Supplementary material for: Mitochondrial unfolded protein response transcription factor ATFS-1 promotes longevity in a long-lived mitochondrial mutant through activation of stress response pathways
Source: BMC Biol. 2018 Dec 18;16:147. doi: 10.1186/s12915-018-0615-3 (PMC6298126; doi:10.1186/s12915-018-0615-3)
Supplement: Supplementary file 1 — Figure S1. hsp-6 mRNA levels are increased in young adult clk-1, isp-1 and nuo-6 worms and correlated with atfs-1 mRNA levels. Figure S2. Activation of the mitochondrial unfolded protein response in long-lived mitochondrial mutants is dependent on atfs-1. Figure S3. Treatment with antioxidants does not prevent activation of the mitochondrial unfolded protein response in nuo-6 mutants. Figure S5. ATFS-1 is required for development to adulthood in clk-1 and isp-1 worms. Figure S6. nuo-6 worms requires UBL-5 and DVE-1 to develop to adulthood. Figure S7. haf-1 is not required for the longevity of the long-lived mitochondrial mutant nuo-6. Figure S8. Effect of knocking down components of the mitochondrial unfolded protein response on activation of the cytoplasmic unfolded protein response. Figure S9. Mitochondrial unfolded protein response is activated in sod-2 mutants but not required for their longevity. Figure S10. Restoring atfs-1 expression during adulthood reverts lifespan to nuo-6. Figure S11. Disruption of atfs-1 decreases levels of reactive oxygen species (ROS). Figure S12. Activation of cytosolic unfolded protein response in atfs-1 mutants is HSF-1-dependent and DAF-16-independent. Figure S13. ATFS-1 is required for the activation of stress response pathways in nuo-6 worms. Figure S14. The expression of antioxidant defense genes is not significantly modulated by atfs-1 deletion. Figure S15. HIF-1-regulated gene expression changes in nuo-6 mitochondrial mutant. Figure S16. Reactome enrichment for genes upregulated in nuo-6 mutants and reverted to wild-type in nuo-6;hif-1 double mutants. Figure S17. Reactome enrichment for genes downregulated altered in nuo-6 mutants and reverted to wild-type in nuo-6;hif-1 double mutants. Figure S18. Loss of atfs-1 decreases nuclear localization of DAF-16. Figure S19. ATFS-1-regulated gene expression changes in nuo-6 mitochondrial mutant. Figure S20. Reactome enrichment for genes upregulated in nuo-6 mutants and reverted to wild [file 12915_2018_615_MOESM1_ESM.pdf]

Additional files for:

Mitochondrial unfolded protein response transcription factor ATFS-1 promotes longevity in a long-lived mitochondrial mutant through activation of stress response pathways

Ziyun Wu*, Megan M. Senchuk*, Dylan J. Dues, Benjamin K. Johnson, Jason F. Cooper, Leira Lew, Emily Machiela, Claire E. Schaar, Heather DeJonge, T. Keith Blackwell, Jeremy M. Van Raamsdonk

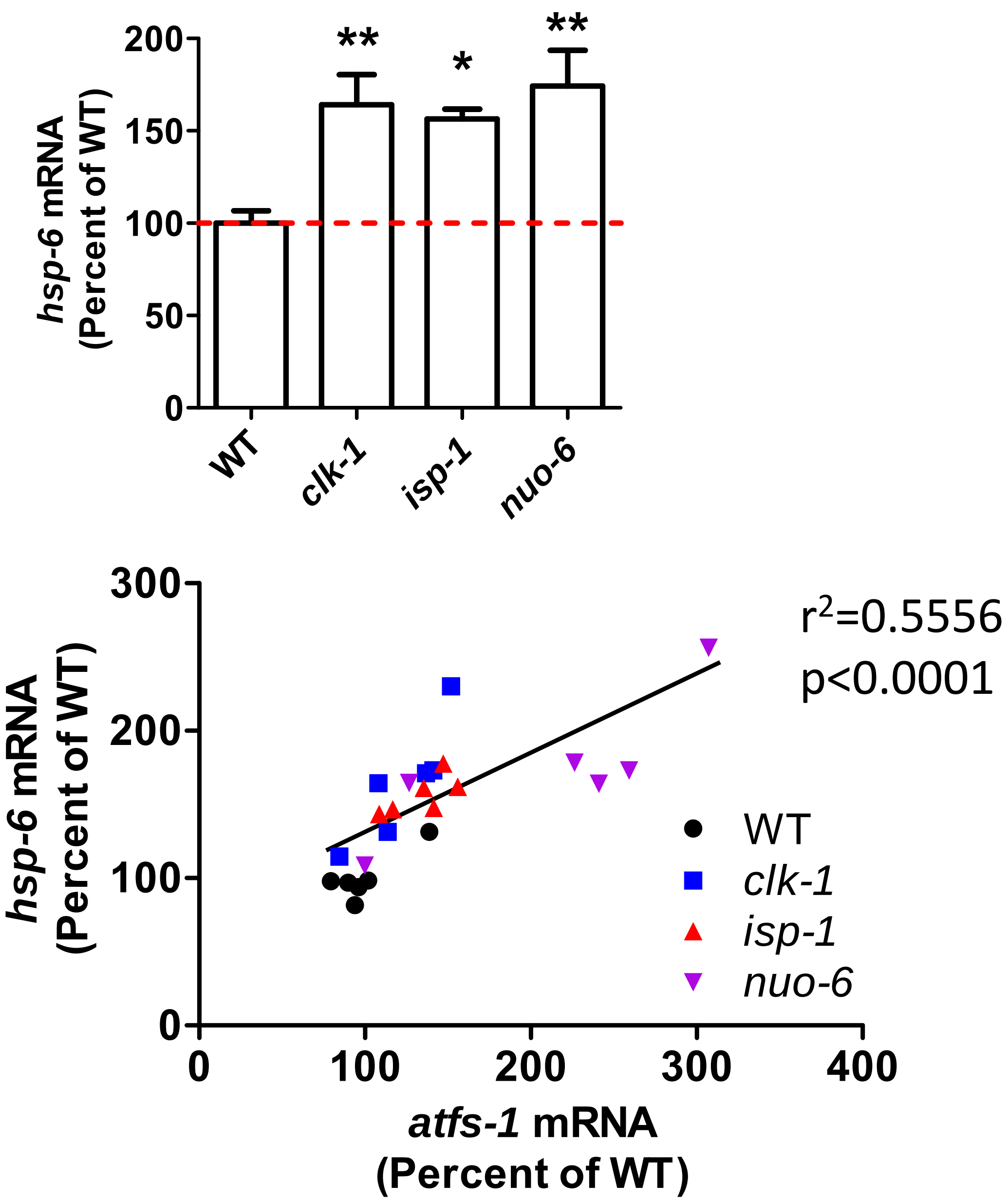

Figure S1. *hsp-6* mRNA levels are increased in young adult *clk-1, isp-1* and *nuo-6* worms and correlated with *atfs-1* mRNA levels. *atfs-1* and *hsp-6* mRNA levels in wild-type, *clk-1, isp-1* and *nuo-6* worms were obtained from RNA sequencing data (6 biological replicate per strain). **A.** *clk-1, isp-1* and *nuo-6* mutants all show increased *hsp-6* mRNA levels on day 1 of adulthood. **B.** There is a significant, positive correlation between *hsp-6* mRNA levels and *atfs-1* mRNA levels.

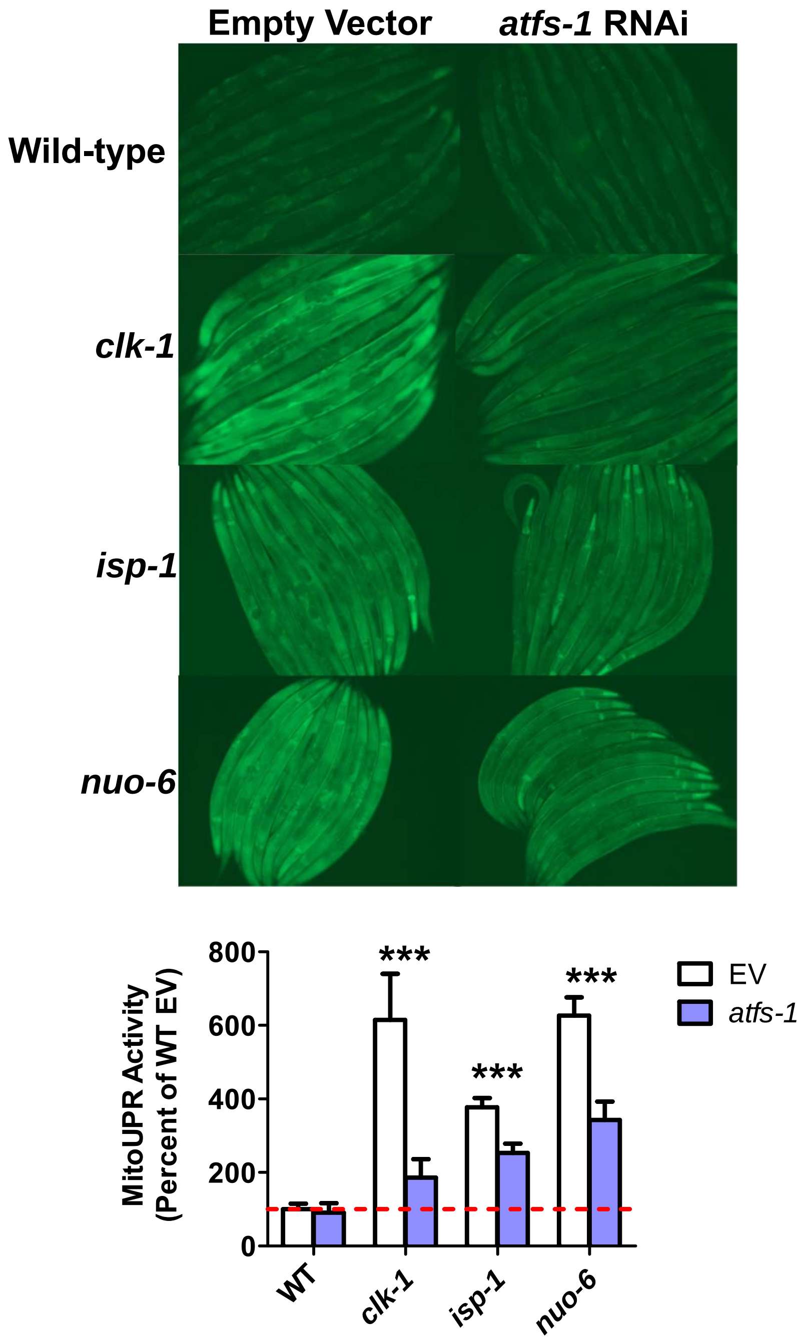

Figure S2. Activation of the mitochondrial unfolded protein response in long-lived mitochondrial mutants is dependent on *atfs-1*. Worms were treated with EV (empty vector) or *atfs-1* RNAi beginning at the L4 stage of development. Mitochondrial unfolded protein response activity was measured by the level of fluorescence from the *Phsp-6::GFP* reporter. Fluorescence was measured after 2 days on RNAi. In *clk-1, isp-1* and *nuo-6* worms, *Phsp-6::GFP* reporter fluorescence was increased compared to wild-type worms and was decreased by treatment with *atfs-1* RNAi. This indicates that ATFS-1 is required for activation of the mitoUPR in these strains. Error bars indicate SEM. ***p<0.0001.

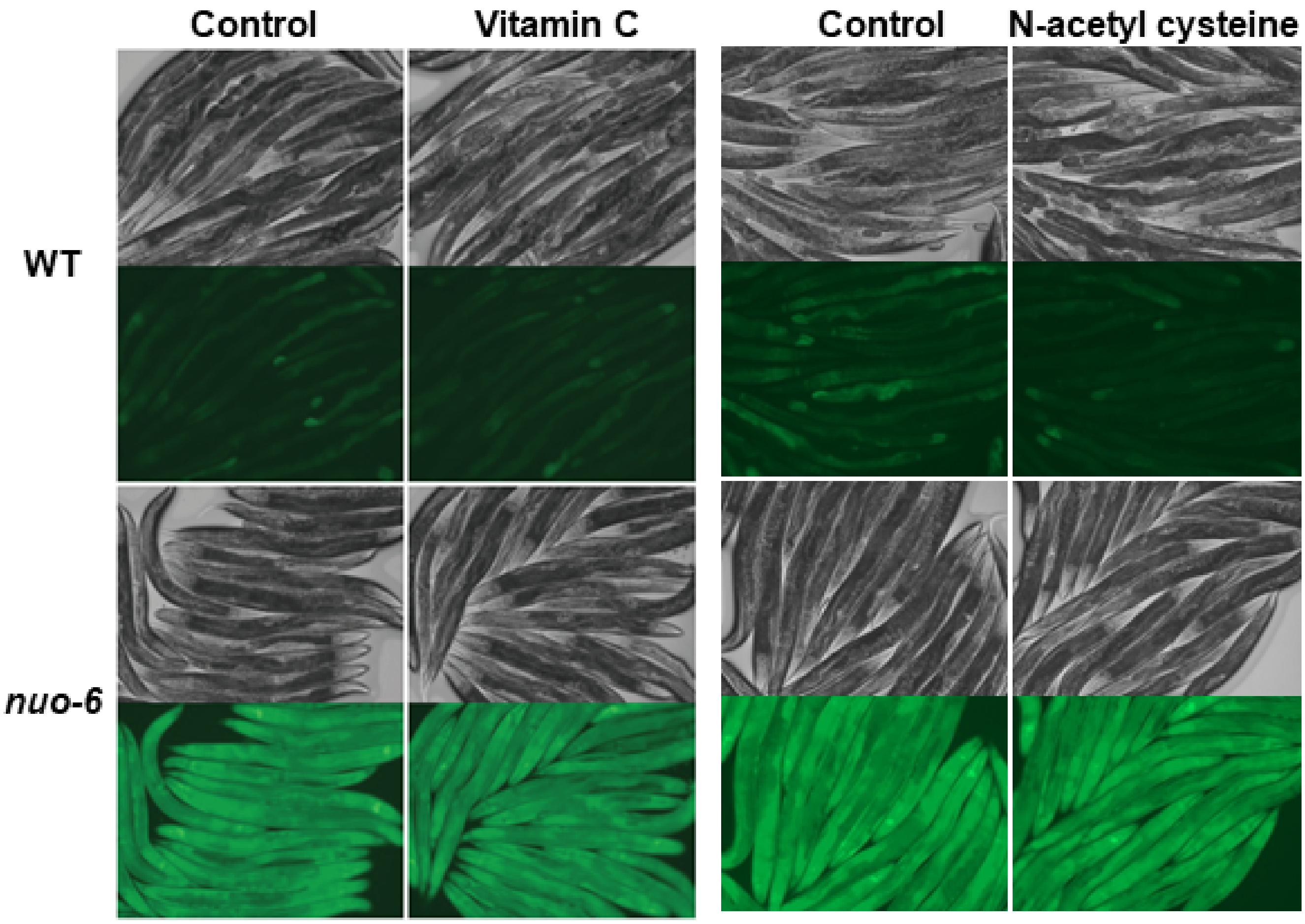

Figure S3. Treatment with antioxidants does not prevent activation of the mitochondrial unfolded protein response in *nuo-6* mutants. Activation of the mitochondrial unfolded protein response was measured by examining the fluorescence of a *Phsp-6::GFP* reporter strain. In both wild-type and *nuo-6* worms treatment with 10 mM Vitamin C or 10 mM N-acetyl cysteine did not decrease fluorescence. This suggests that elevated levels of ROS in *nuo-6* are not required for the activation of the mitochondrial unfolded protein response.

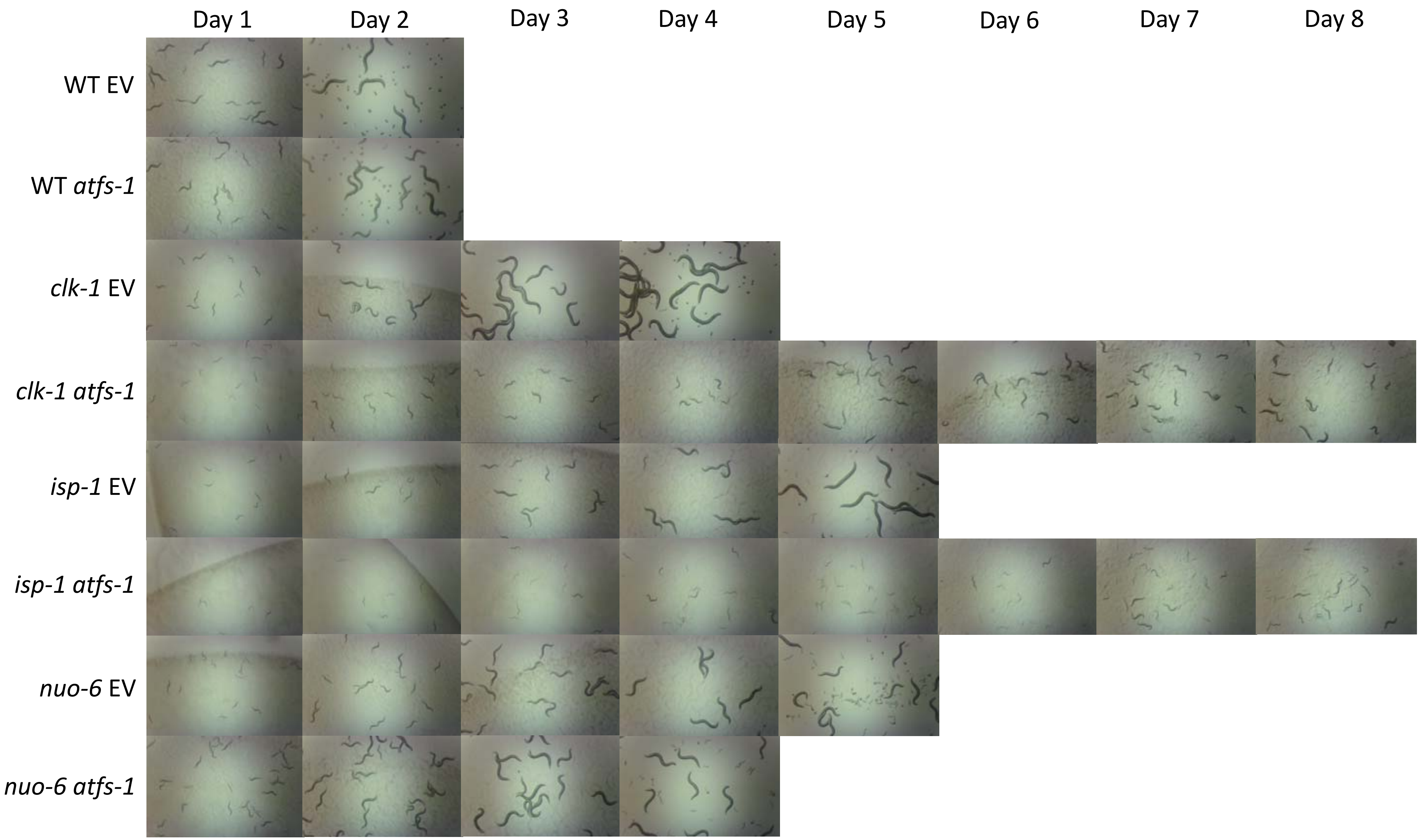

Figure S4. ATFS-1 is required for development to adulthood in *clk-1* and *isp-1* worms. Worms were grown on *atfs-1* RNAi or empty vector beginning at the parental L4 stage. The development of the F1 progeny was monitored daily. While *atfs-1* RNAi does not markedly affect the development of wild-type worms, *clk-1* and *isp-1* worms fail to develop to adulthood on *atfs-1* RNAi. In contrast, *atfs-1* RNAi accelerates the development of *nuo-6* worms.

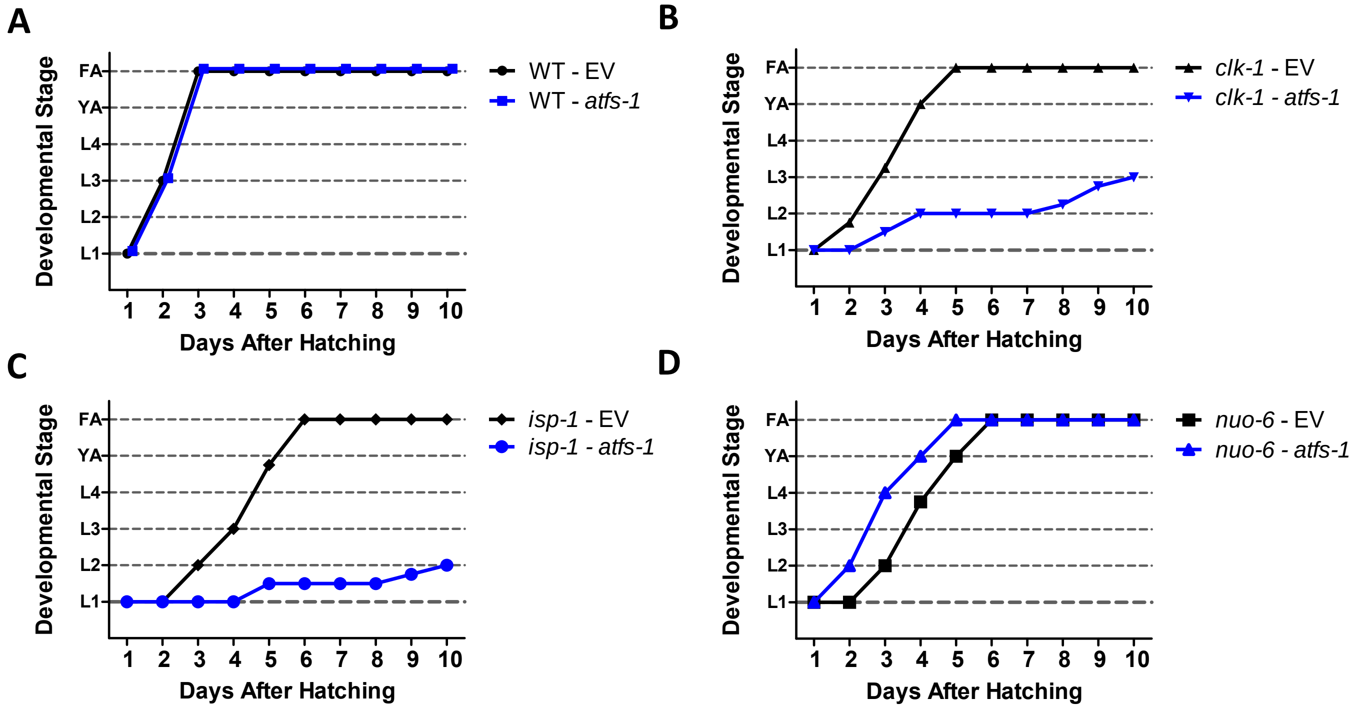

Figure S5. ATFS-1 is required for development to adulthood in *clk-1* and *isp-1* worms. Worms were grown on *atfs-1* RNAi or empty vector beginning at the parental L4 stage. The development of the F1 progeny was monitored daily. **A.** *atfs-1* RNAi does not markedly affect the development of wild-type worms. **B.** *clk-1* worms fail to develop to adulthood on *atfs-1* RNAi. On average worms reach the L3 stage of development. **C.** Similarly, *isp-1* worms also fail to develop to adulthood on *atfs-1* RNAi. These worms typically arrest at the L2 stage. **D.** In contrast, *atfs-1* RNAi accelerates the development of *nuo-6* worms. *atfs-1* RNAi-treated *nuo-6* worms develop approximately one day faster than *nuo-6* worms on empty vector. Two replicates were performed. Images can be found in supplemental Fig. S3.

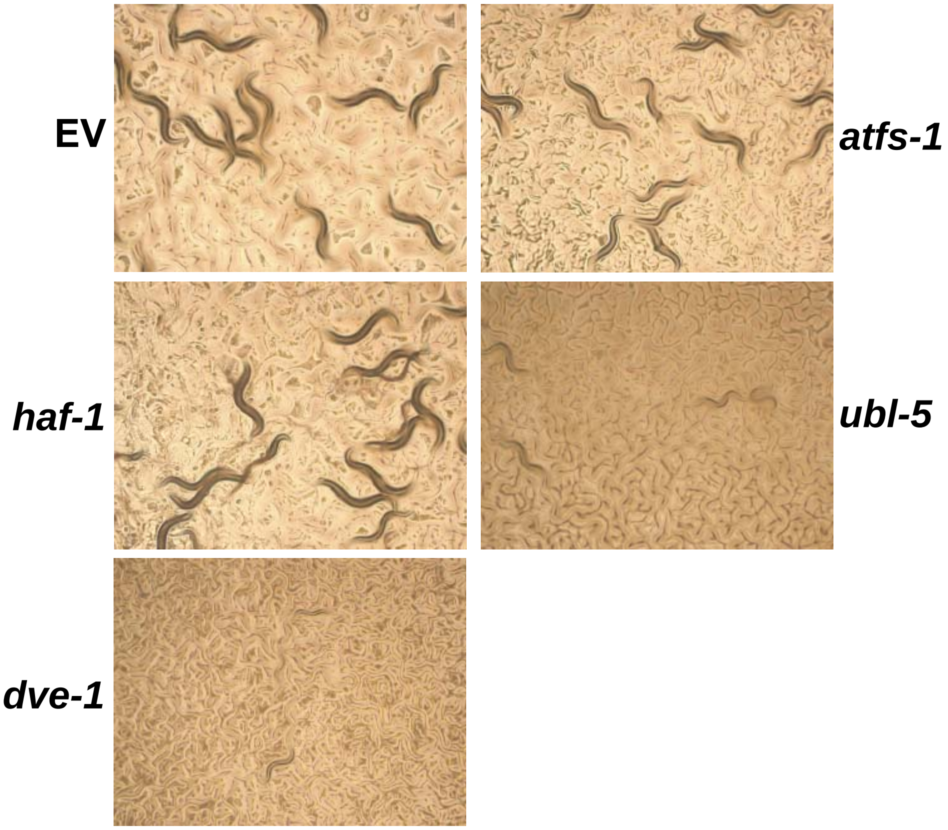

Figure S6. *nuo-6* worms requires UBL-5 and DVE-1 to develop to adulthood*.* *nuo-6* worms were treated with RNAi to disrupt different components of the mitochondrial unfolded protein response (mitoUPR). RNAi treatment was begun in the parental L4 stage and the development of the progeny was monitored. Worms grown on *ubl-5* or *dve-1* RNAi failed to develop to adulthood. Images were taken on day 7 after hatching.

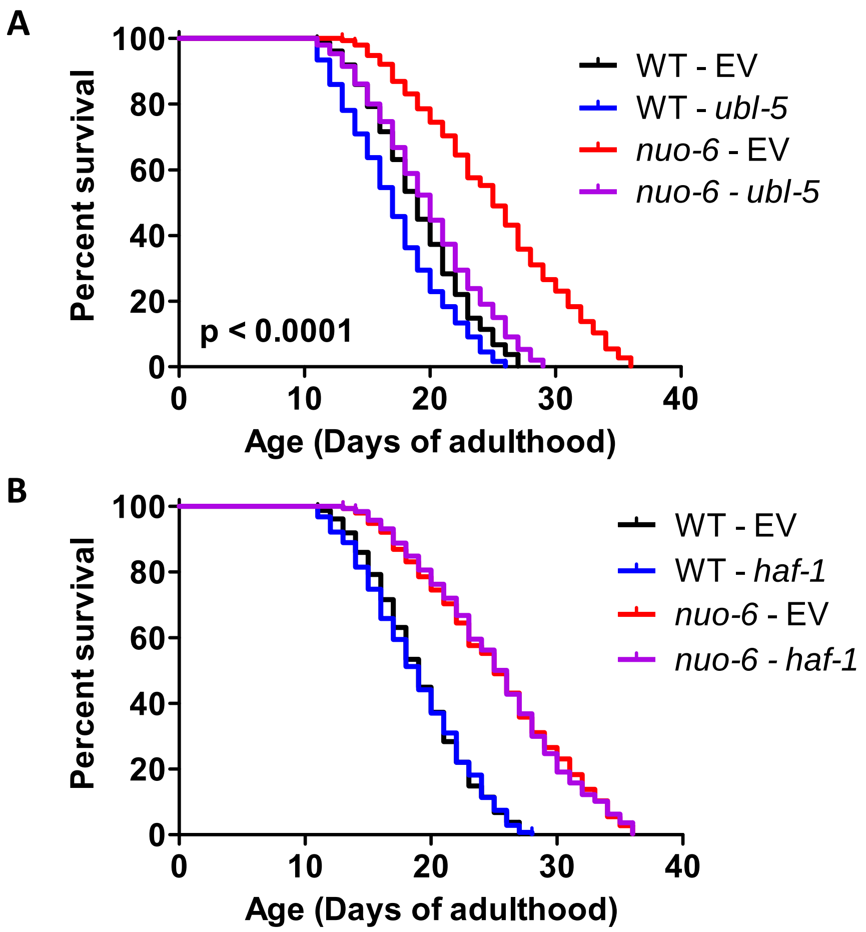

Figure S7. *haf-1* is not required for the longevity of the long-lived mitochondrial mutant *nuo-6*. To test the role of other components of the mitochondrial unfolded protein response (mitoUPR) in the lifespan of *nuo-6* mutants, *ubl-5* and *haf-1* were knocked down using RNAi beginning the parental generation at the L4 stage. Under these conditions, *nuo-6* worms did not develop past the L4 stage on *ubl-5* RNAi. **A.** Examination of the survival of L4 worms revealed that *ubl-5* RNAi decreased L4 survival in *nuo-6* mutants. *ubl-5* RNAi also mildly decreased lifespan in wild-type worms. **B.** Both wild-type and *nuo-6* worms develop to adulthood on *haf-1* RNAi. *haf-1* RNAi did not affect the longevity of wild-type or *nuo-6* worms. L4 survival and lifespan was measured from hatching. p value indicates difference between *nuo-6* EV (red line) and *nuo-6 + ubl-5* RNAi (purple line).

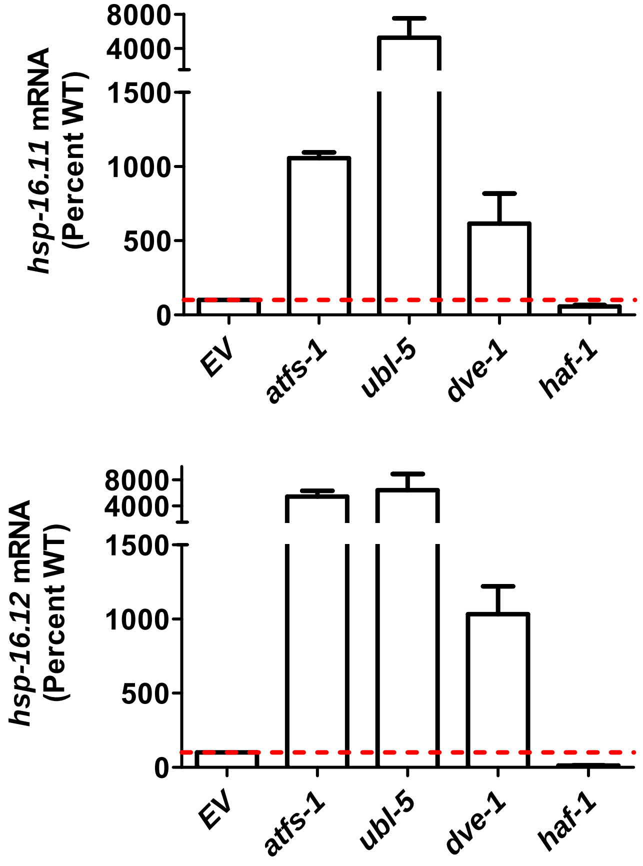

Figure S8. Effect of knocking down components of the mitochondrial unfolded protein response on activation of the cytoplasmic unfolded protein response. The expression of different components of the mitochondrial unfolded protein response were decreased using RNAi in wild-type worms. Levels of the cytosolic unfolded protein response targets *hsp-16.11* and *hsp-16.12* were measured using quantitative real-time RT-PCR. Decreasing the levels of *atfs-1, ubl-5* or *dve-1* resulted in increased levels of both *hsp-16.11* and *hsp-16.2.* In contrast, decreasing levels of *haf-1* had no significant effect on the expression of either gene. Error bars indicate SEM.

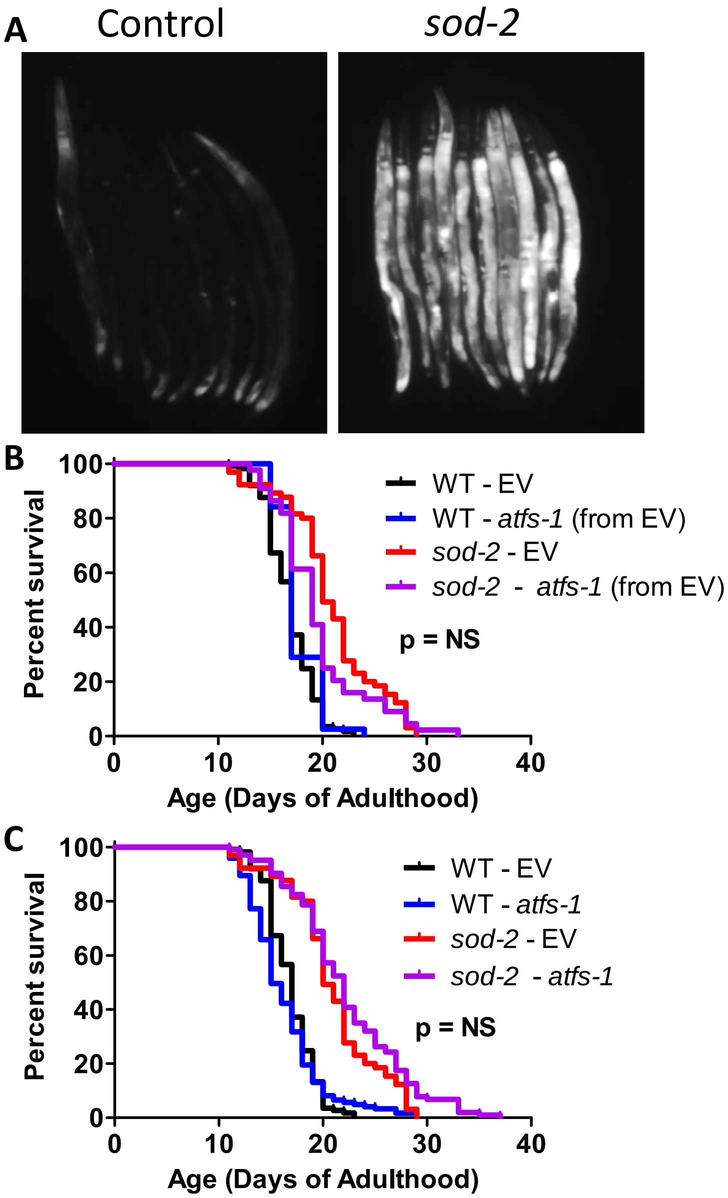

Figure S9. Mitochondrial unfolded protein response is activated in *sod-2* mutants but not required for their longevity. *sod-2* worms were crossed with the mitoUPR reporter strain *Phsp-6::GFP.* **A.** *sod-2;Phsp-6::GFP* worms showed increased fluorescence compared to *Phsp-6::GFP* indicating that the mitoUPR is activated. Preventing the activation of the mitoUPR through *atfs-1* RNAi during adulthood only (**B**) or throughout development and adulthood (**C**) does not decrease the lifespan of *sod-2* worms. This suggests that the activation of the mitoUPR does not contribute to their longevity. Significance between red and purple lines is indicated.

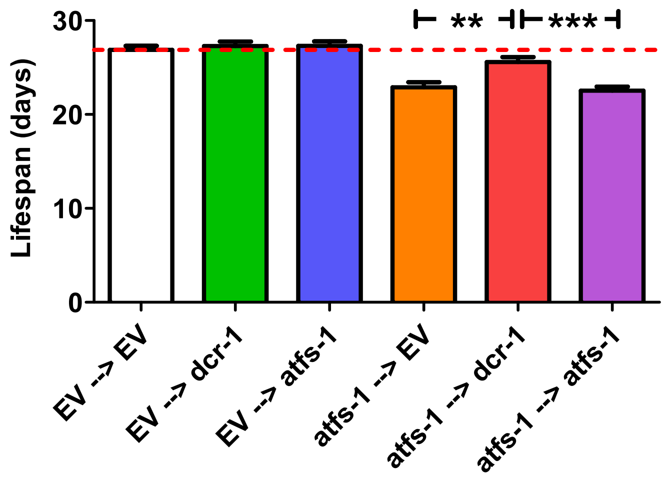

Figure S10. Restoring *atfs-1* expression during adulthood reverts lifespan to *nuo-6.* A. To explore the timing requirements for the role of ATFS-1 in *nuo-6* longevity and the activation of the mitoUPR, *nuo-6* worms were treated with RNAi against *atfs-1* for development only (*atfs-1* → *dcr-1*), adulthood only (EV → *atfs-1*) or for both development and adulthood (*atfs-1* → *atfs-1*). Knocking down *atfs-1* levels only during adulthood (EV → *atfs-1*) or only during development (*atfs-1* → *dcr-1*) does not affect *nuo-6* lifespan. In contrast, *atfs-1* RNAi applied during both development and adulthood (*atfs-1* → *atfs-1*) decreased *nuo-6* longevity. Worms treated with the *atfs-1* → EV paradigm also show a decrease in lifespan presumably because knockdown of *atfs-1* continues during adulthood. This is in contrast to worms treated with the *atfs-1* → *dcr-1* paradigm. Error bars indicate SEM. **p<0.01, ***p<0.001.

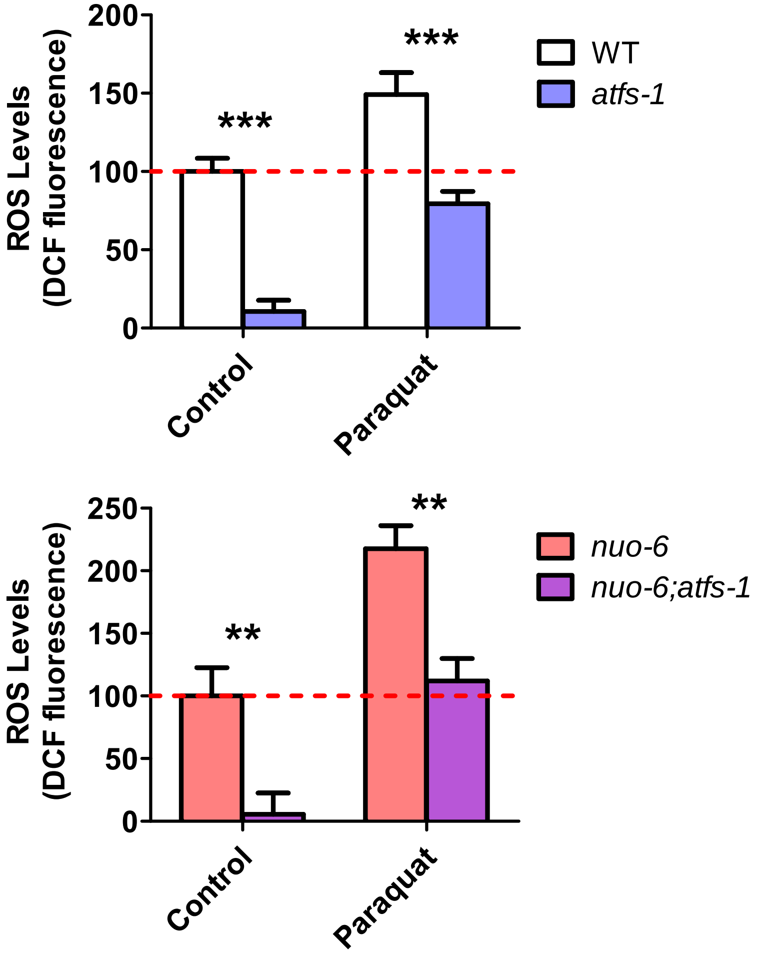

Figure S11. Disruption of *atfs-1* decreases levels of reactive oxygen species (ROS). ROS levels were measured using the ROS-sensitive dye dichloroluorescein (DCF). In both wild-type (WT) and *nuo-6* worms, deletion of *atfs-1* resulted in markedly decreased levels of ROS. As a control, worms were treated with the superoxide-generating compound paraquat. As expected, paraquat treated worms show increased levels of DCF fluorescence. Error bars indicate SEM. **p<0.01, ***p<0.001.

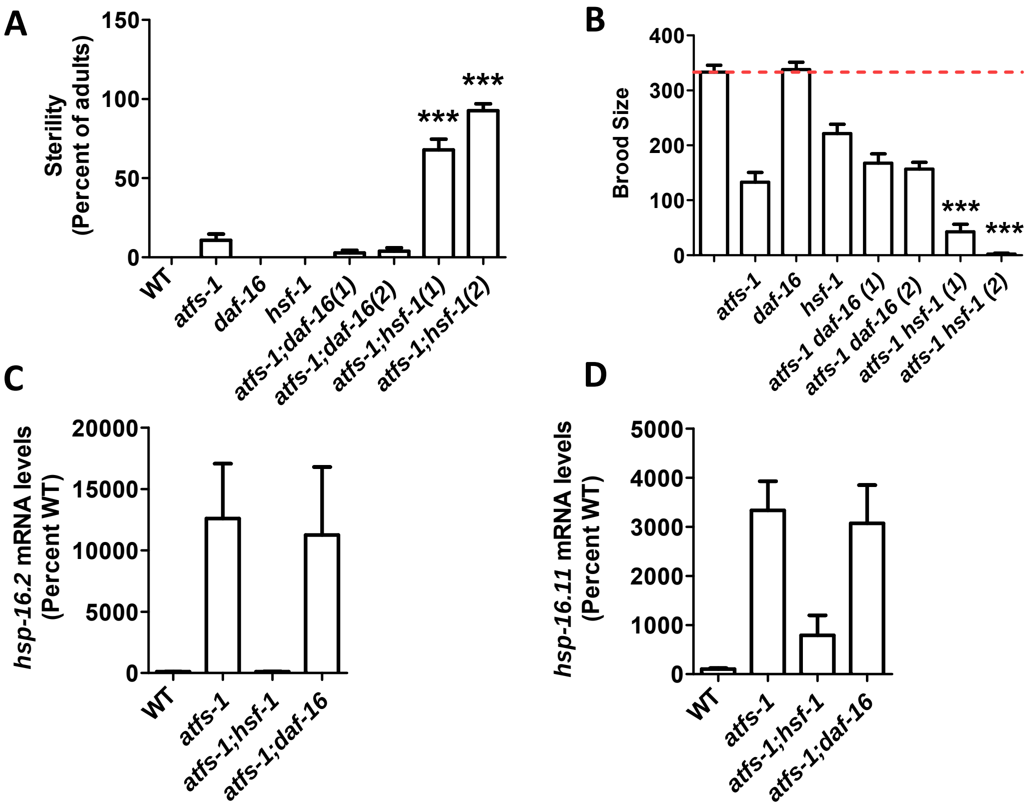

Figure S12. Activation of cytosolic unfolded protein response in *atfs-1* mutants is HSF-1-dependent and DAF-16-independent. To explore the mechanism of *hsp-16.2* upregulation in *atfs-1* deletion mutants, we crossed *atfs-1* worms to *hsf-1* and *daf-16* mutants. A large percentage of *atfs-1;hsf-1* double mutants are sterile (**A**) and those that produce progeny have a markedly decreased brood size (**B**). This suggests that the activation of the cytosolic unfolded protein response in *atfs-1* mutant is important for the health and fertility of these worms. Examining which transcription factors are required for the upregulation of the cytosolic unfolded protein response, we find that the increased expression of *hsp-16.2* (**C**) and *hsp-16.11* (**D**) is dependent on HSF-1 but not DAF-16. Error bars indicate SEM. ***p<0.001.

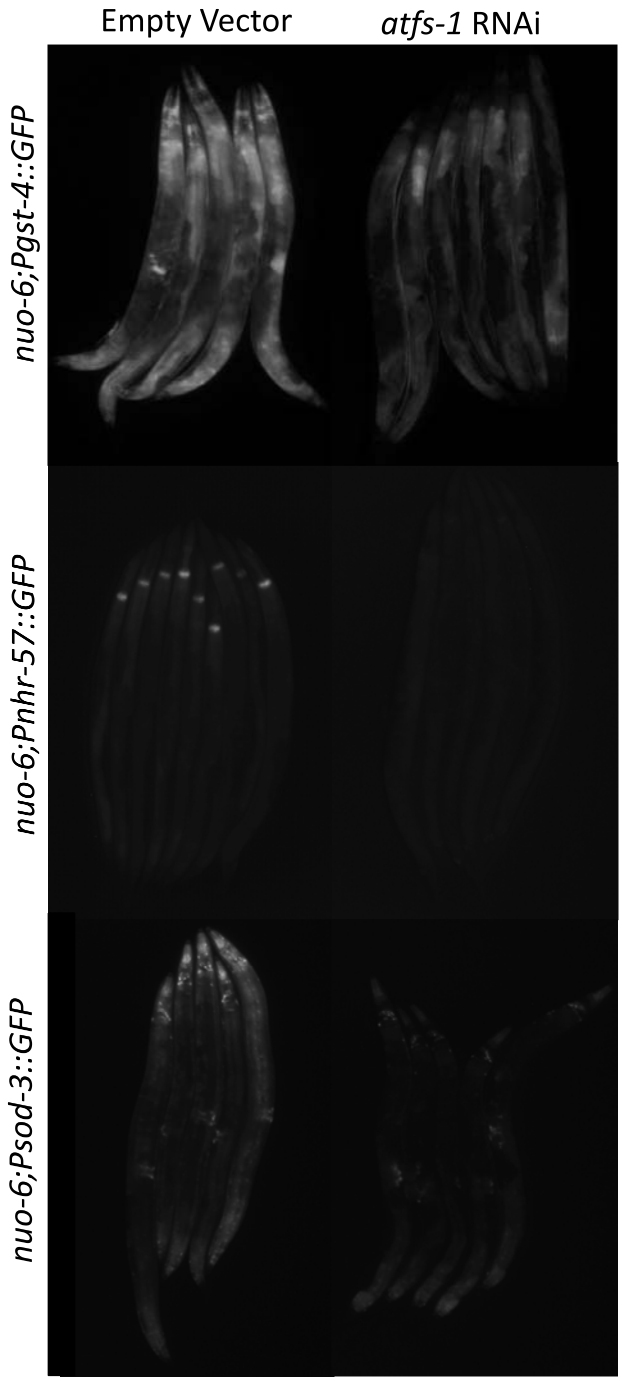

Figure S13. ATFS-1 is required for the activation of stress response pathways in *nuo-6* worms. *nuo-6* worms have increased fluorescence from reporter strains for the SKN-1-mediated oxidative stress response (*Pgst-4::GFP*), the HIF-1-mediated hypoxia response (*Pnhr-57::GFP*), and the DAF-16-mediated stress response (*Psod-3::GFP*). In each case, treatment with *atfs-1* RNAi results in decreased fluorescence from the reporter strain.

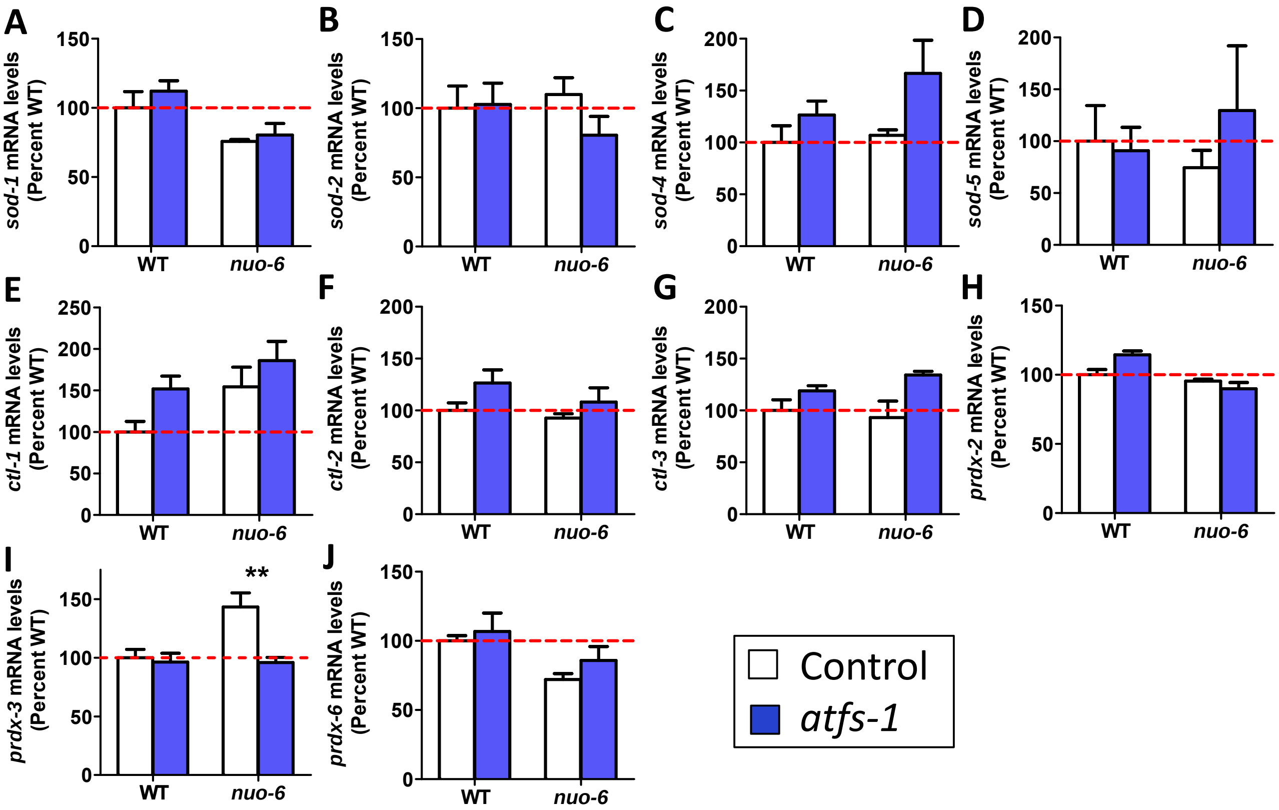

Figure S14. The expression of antioxidant defense genes is not significantly modulated by *atfs-1* deletion. Error bars indicate SEM.

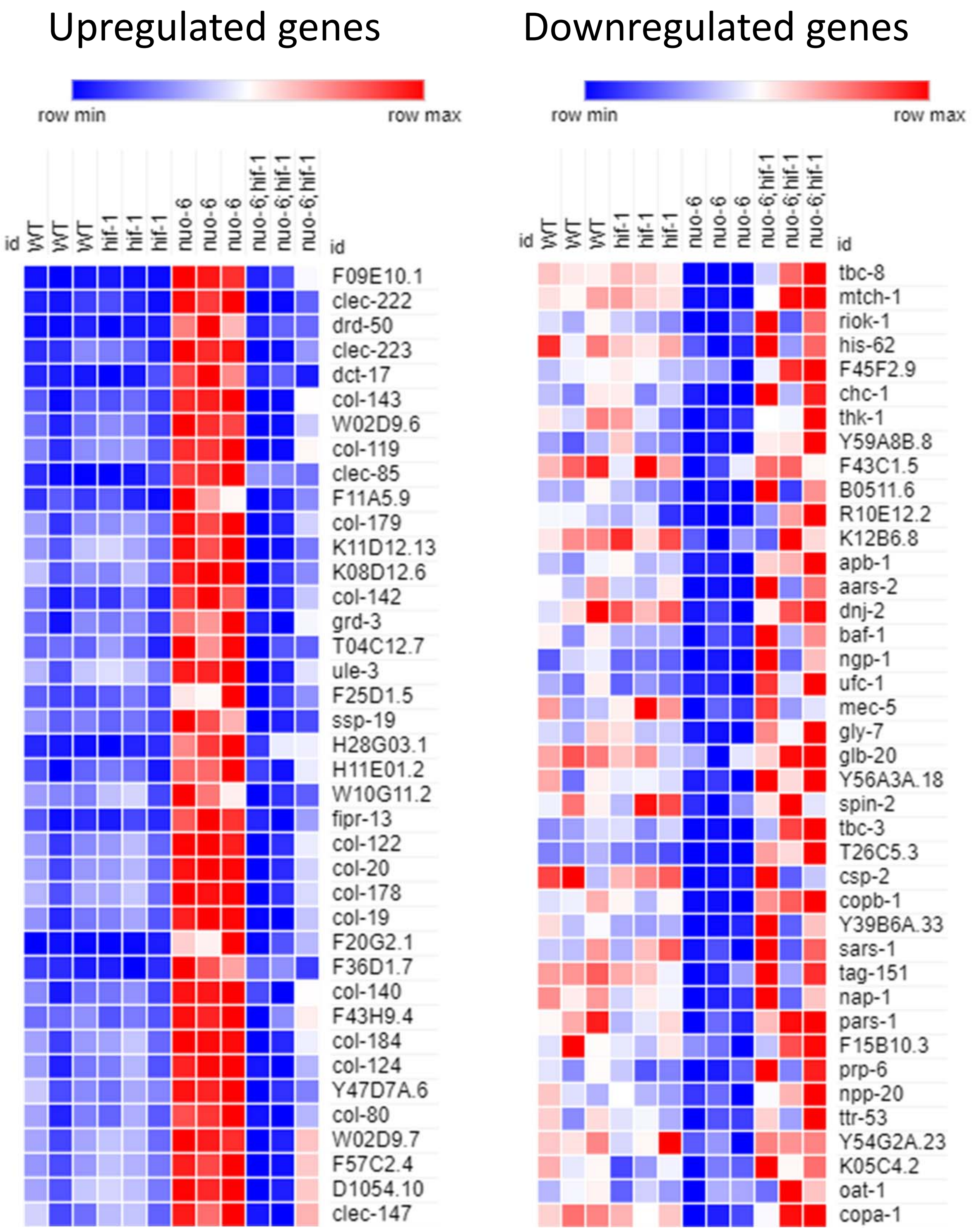

Figure S15. HIF-1-regulated gene expression changes in *nuo-6* mitochondrial mutant. Gene expression was quantified using RNAseq with three biological replicates per strain. Numerous genes that are either upregulated or downregulated in *nuo-6* mutants are restored to wild-type by the loss of *hif-1* but unaffected by the *hif-1* mutation in a wild-type background.

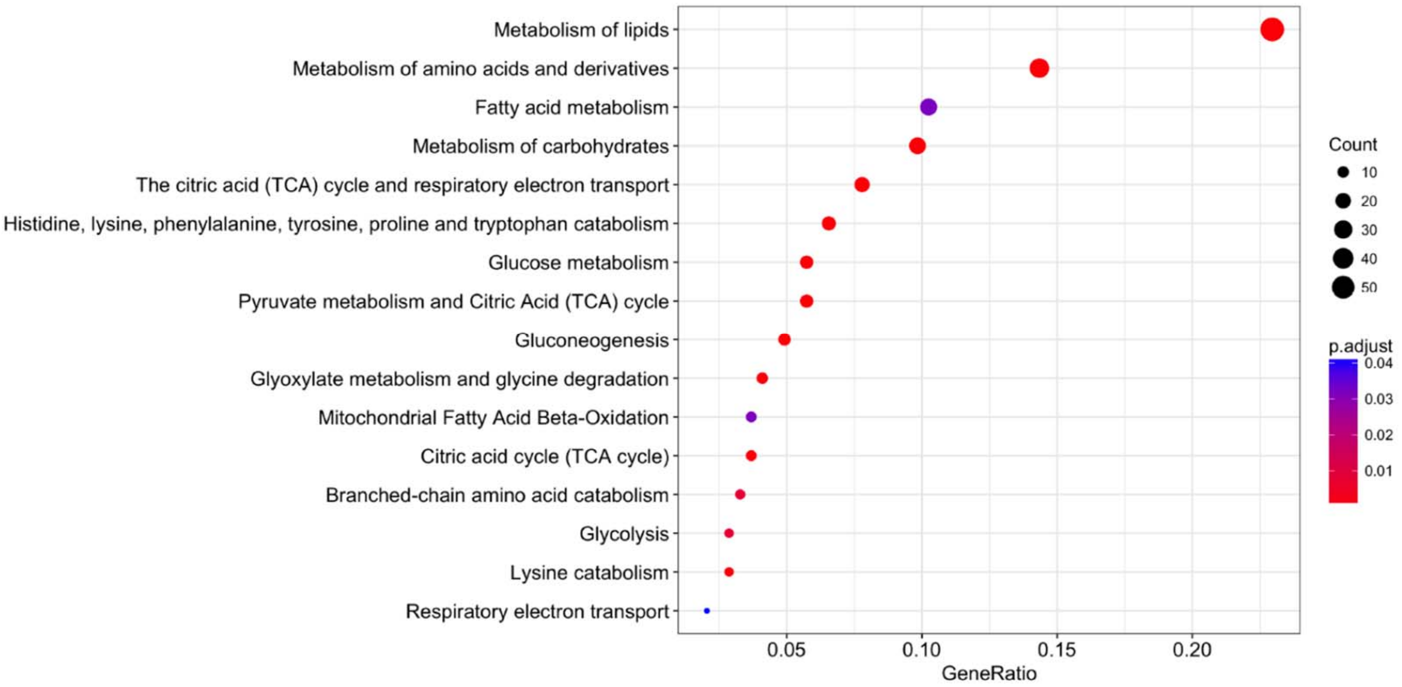

Figure S16. Reactome enrichment for genes upregulated in *nuo-6* mutants and reverted to wild-type in *nuo-6;hif-1* double mutants. The size of the circle indicates the number of genes in each category. The color of the circles indicates the level of significance. Gene Ratio is the number of genes in a category that are significantly upregulated or downregulated divided by the total number of genes in each category.

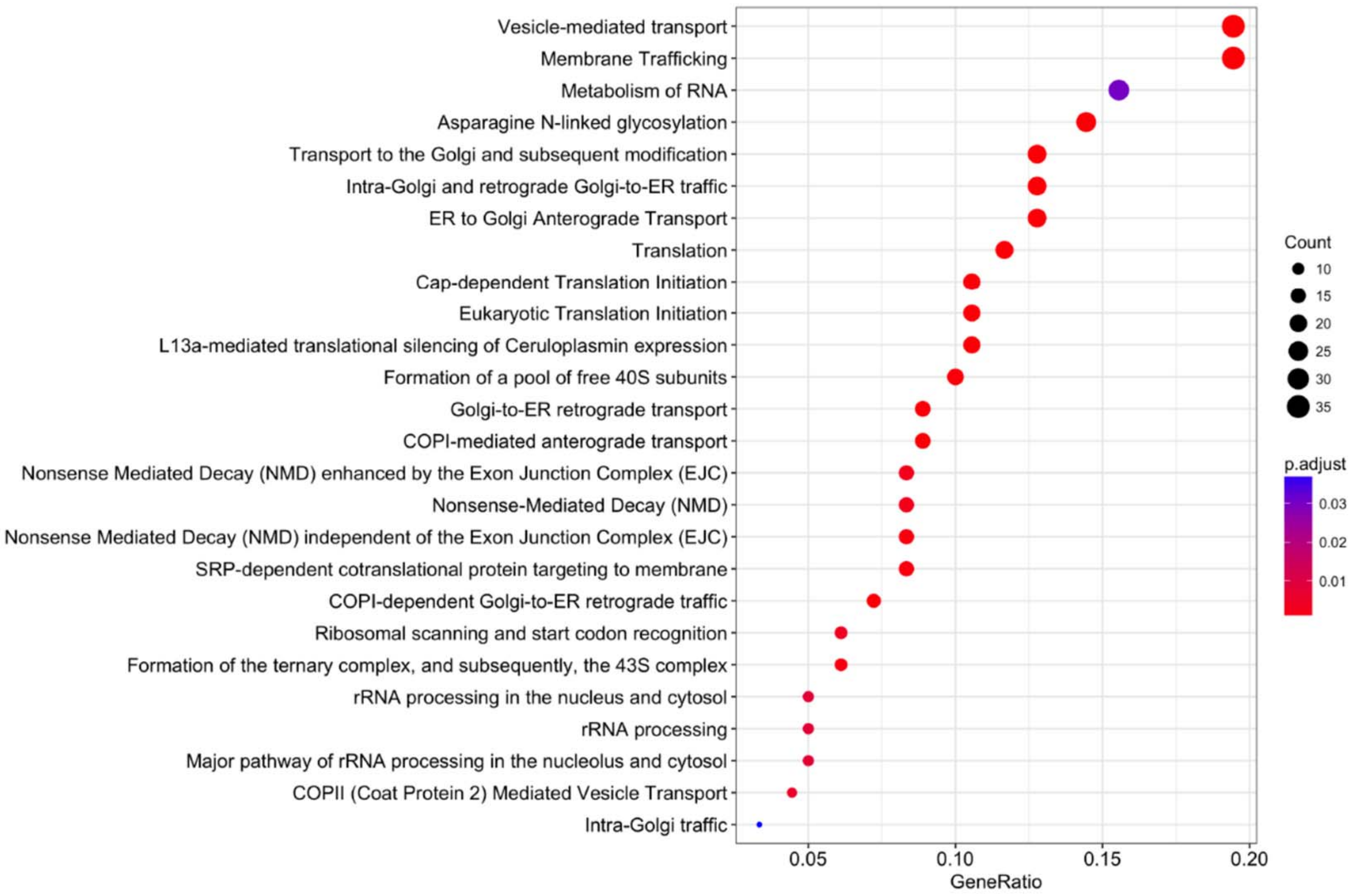

Figure S17. Reactome enrichment for genes downregulated altered in *nuo-6* mutants and reverted to wild-type in *nuo-6;hif-1* double mutants. The size of the circle indicates the number of genes in each category. The color of the circles indicates the level of significance. Gene Ratio is the number of genes in a category that are significantly upregulated or downregulated divided by the total number of genes in each category.

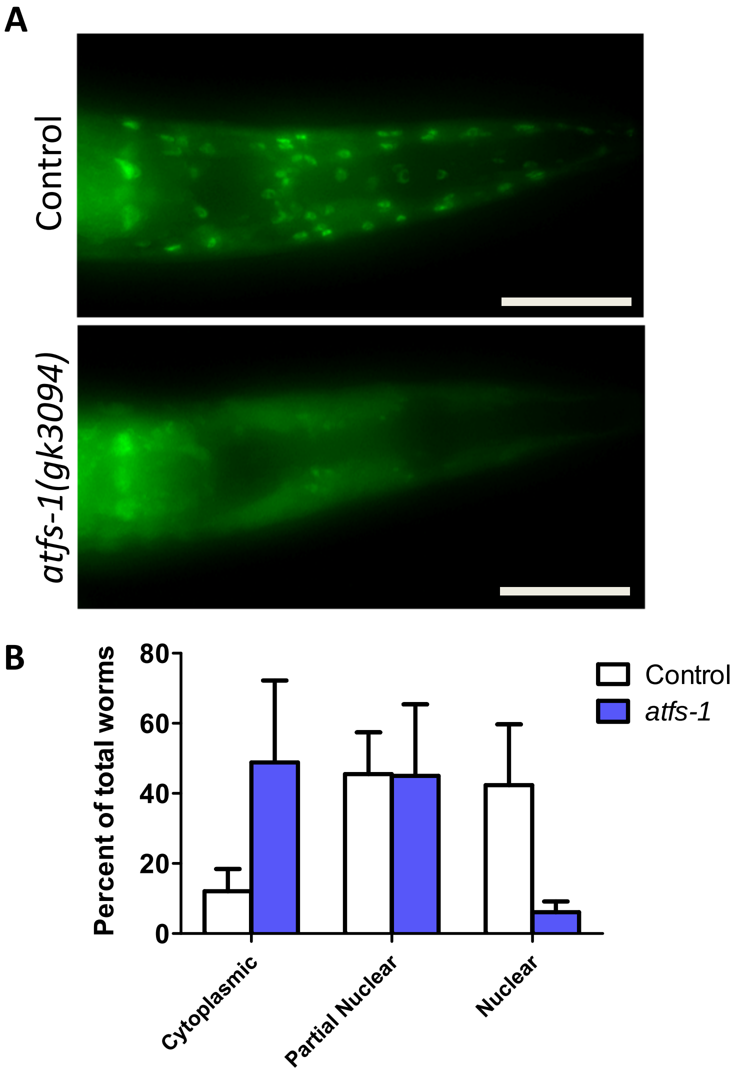

Figure S18. Loss of *atfs-1* decreases nuclear localization of DAF-16. The nuclear localization of DAF-16 was monitored using *zIs356[Pdaf-16::DAF-16::GFP]* worms (control) after exposure to heat at 35° C. In the presence of the *atfs-1(gk3094)* deletion mutation, the nuclear localization of DAF-16 was decreased compared to wild-type. **A.** Representative images. **B.** Quantification of percentage of worms exhibiting cytoplasmic, partial nuclear or nuclear localization of DAF-16:GFP. Scale bar indicates 0.05 mm.

Upregulated genes

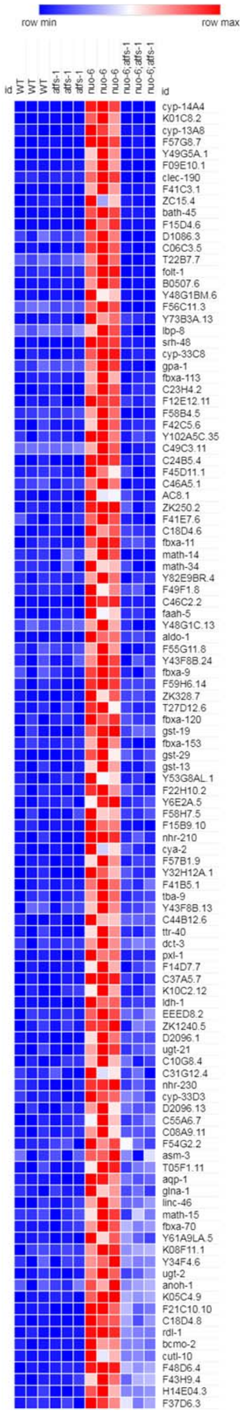

Downregulated genes

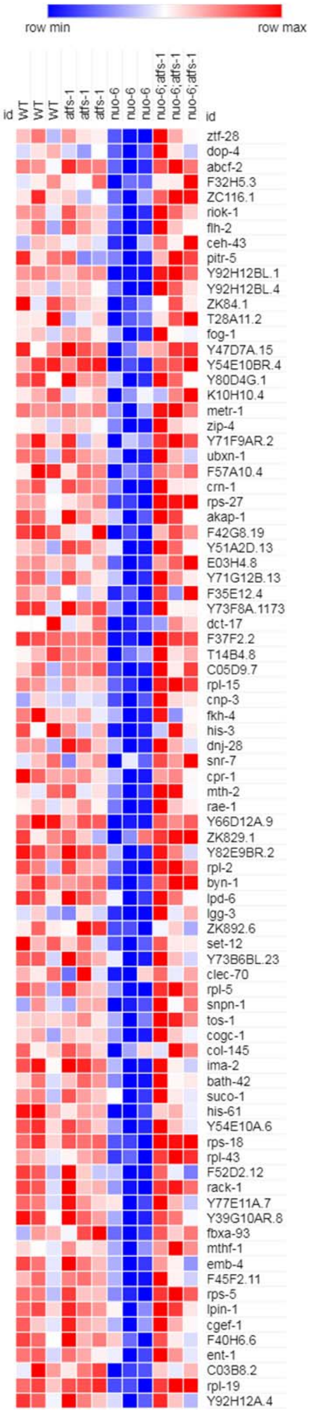

Figure S19. ATFS-1-regulated gene expression changes in *nuo-6* mitochondrial mutant. Gene expression was quantified using RNAseq with three biological replicates per strain. Numerous genes that are either upregulated or downregulated in *nuo-6* mutants are restored to wild-type by the loss of *atfs-1* but unaffected by the *atfs-1* mutation in a wild-type background.

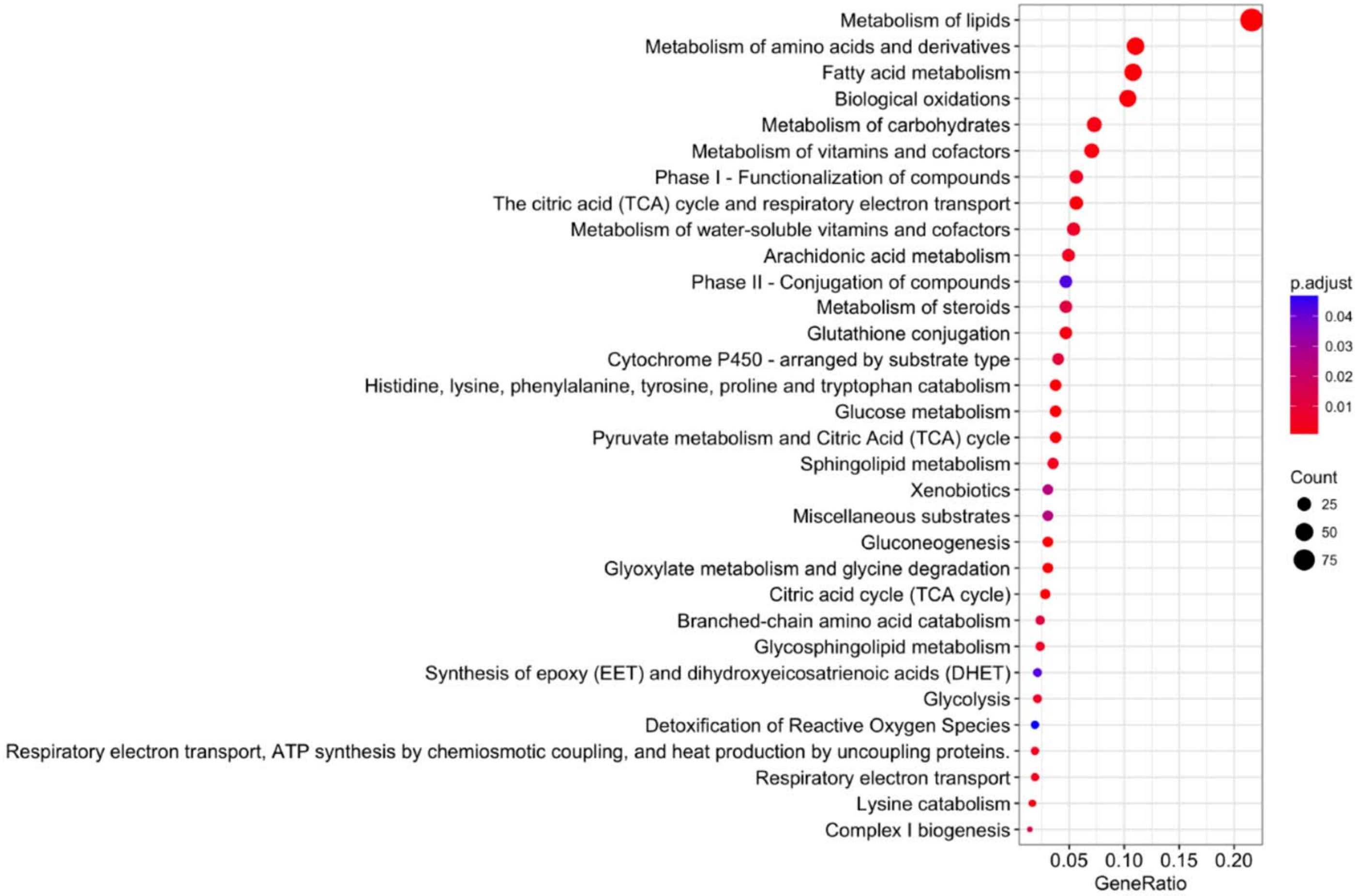

Figure S20. Reactome enrichment for genes upregulated in *nuo-6* mutants and reverted to wild-type in *nuo-6;atfs-1* double mutants. The size of the circle indicates the number of genes in each category. The color of the circles indicates the level of significance. Gene Ratio is the number of genes in a category that are significantly upregulated or downregulated divided by the total number of genes in each category.

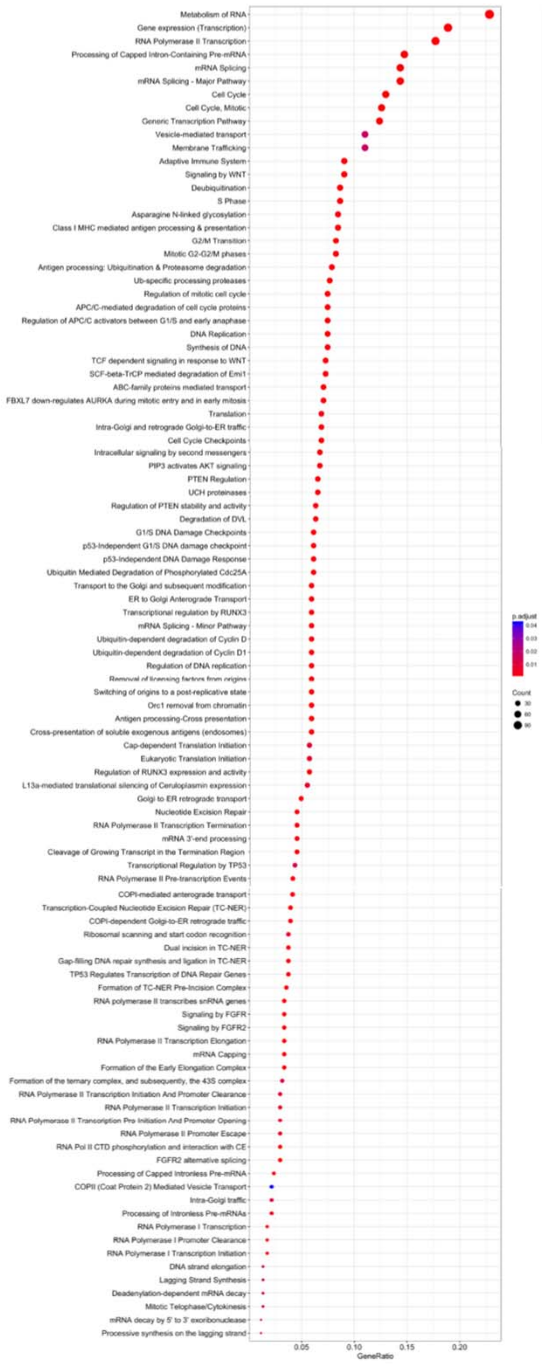

Figure S21. Reactome enrichment for genes downregulated in *nuo-6* mutants and reverted to wild-type in *nuo-6;atfs-1* double mutants. The size of the circle indicates the number of genes in each category. The color of the circles indicates the level of significance. Gene Ratio is the number of genes in a category that are significantly upregulated or downregulated divided by the total number of genes in each category.

A

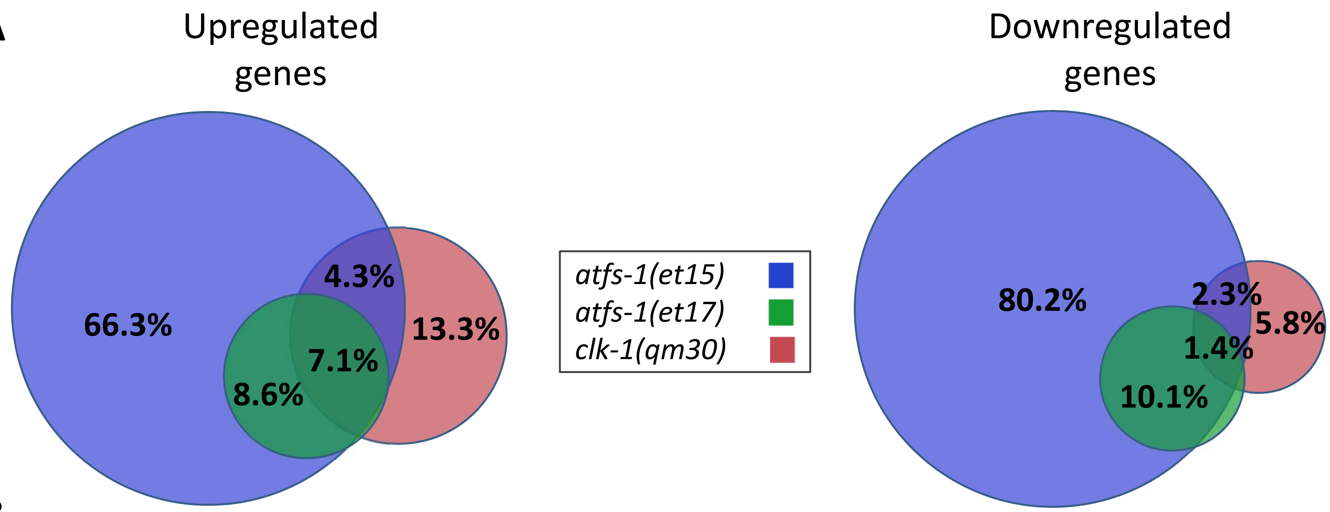

B

	Strain	Number of significantly modulated genes	Number of overlapping genes	Expected Number of overlapping genes	Chi Square	Significance
Genes upregulated in *clk-1* (838)	*atfs-1(et15)*	2911	385	174	273	P<0.00001
	atfs-1(et17)	544	244	32	1464	P<0.00001
Genes downregulated in *clk-1* (336)	*atfs-1(et15)*	3316	129	79	32	P<0.00001
	atfs-1(et17)	414	51	10	174	P<0.00001

Figure S22. *atfs-1* gain-of-function mutants show a highly significant overlap in gene expression changes with *clk-1* mutants. A. Venn diagrams indicate the overlap in differentially expressed genes between the two *atfs-1* gain-of-function mutants (*et15* and *et17*) and *clk-1(qm30)* worms. Percentages indicate the percent of all of the genes that are differentially expressed in one of the three mutants. **B.** For both upregulated genes and downregulated genes the degree of overlap is highly significant.

A

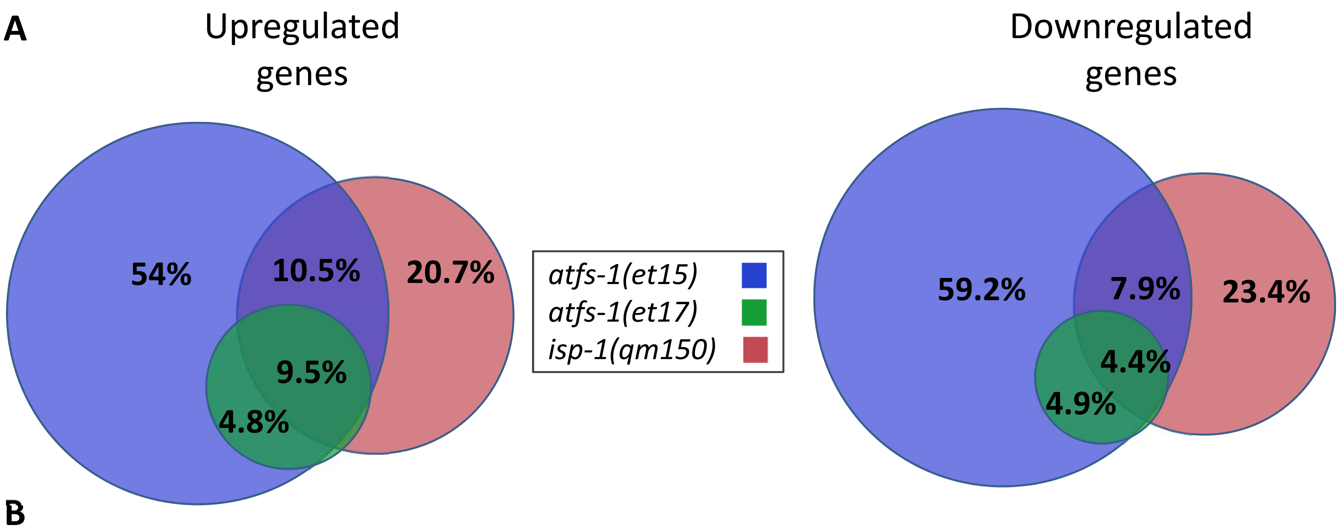

B

	Strain	Number of significantly modulated genes	Number of overlapping genes	Expected Number of overlapping genes	Chi Square	Significance
**Genes upregulated in *isp-1* (1511)**	*atfs-1(et15)*	2911	738	313	644	P<0.00001
	atfs-1(et17)	544	358	59	1715	P<0.00001
**Genes downregulated in *isp-1* (1550)**	*atfs-1(et15)*	3316	533	366	85	P<0.00001
	atfs-1(et17)	414	193	46	533	P<0.00001

Figure S23. *atfs-1* gain-of-function mutants show a highly significant overlap in gene expression changes with *isp-1* mutants. A. Venn diagrams indicate the overlap in differentially expressed genes between the two *atfs-1* gain-of-function mutants (*et15* and *et17*) and *isp-1(qm150)* worms. Percentages indicate the percent of all of the genes that are differentially expressed in one of the three mutants. **B.** For both upregulated genes and downregulated genes the degree of overlap is highly significant.

Table S1. ***atfs-1*** **gain-of-function mutants exhibit statistically significant overlap in gene expression changes with** ***daf-2*** **mutants.**

	Strain	Number of significantly modulated genes	Number of overlapping genes	Expected Number of overlapping genes	Chi Square	Significance
**Genes upregulated in *daf-2* mutants (2594)**	*atfs-1(et15)*	2911	868	538	248	P<0.00001
	atfs-1(et17)	544	243	101	247	P<0.00001
**Genes downregulated in *daf-2* mutants (2155)**	*atfs-1(et15)*	3316	1123	509	875	P<0.00001
	atfs-1(et17)	414	260	64	717	P<0.00001
